# Unfamiliarity generates costly aggression in interspecific avian dominance hierarchies

**DOI:** 10.1038/s41467-023-44613-0

**Published:** 2024-01-06

**Authors:** Gavin M. Leighton, Jonathan P. Drury, Jay Small, Eliot T. Miller

**Affiliations:** 1 Department of Biology, SUNY Buffalo State University, Buffalo, NY 14213 USA; 2 Department of Biosciences, Durham University, Durham, United Kingdom; 3 Cornell Lab of Ornithology, Cornell University, Ithaca, NY 14850 USA

**Keywords:** Behavioural ecology, Social evolution, Animal behaviour, Community ecology

## Abstract

Dominance hierarchies often form between species, especially at common feeding locations. Yet, relative to work focused on the factors that maintain stable dominance hierarchies within species, large-scale analyses of interspecific dominance hierarchies have been comparatively rare. Given that interspecific behavioral interference mediates access to resources, these dominance hierarchies likely play an important and understudied role in community assembly and behavioral evolution. To test alternative hypotheses about the formation and maintenance of interspecific dominance hierarchies, we employ an large, participatory science generated dataset of displacements observed at feeders in North America in the non-breeding season. Consistent with the hypothesis that agonistic interference can be an adaptive response to exploitative competition, we find that species with similar niches are more likely to engage in costly aggression over resources. Among interacting species, we find broad support for the hypothesis that familiarity (measured as fine-scale habitat overlap) predicts adherence to the structure of the dominance hierarchy and reduces aggression between species. Our findings suggest that the previously documented agonistic hierarchy in North American birds emerges from species-level adaptations and learned behaviors that result in the avoidance of costly aggression.

## Abstract (web summary)

Although intraspecific dominance hierarchies are common, large scale interspecific dominance hierarchies are unknown. Using data from hundreds of avian species, the authors find that species that are more familiar with each other engage in less aggression and the aggression is resolved more directly.

## Introduction

Interspecific behavioral interactions, such as aggressive contests, are important determinants of ecological and evolutionary processes^1^. Processes such as species range expansion^2^, range limits^3^, character displacement^4,5^, and species recognition^4^ are influenced by aggressive interactions between species. Indeed, rather than being ephemeral cases of mistaken recognition, abundant evidence suggests that aggression between species can arise as an adaptive response to competition for resources^6,7^. Consequently, the evolution and presence of interspecific aggression between species has multifaceted effects on species in certain areas and in certain communities^8,9^.

Interspecific aggression and behavioral interference are critical determinants of resource acquisition for many species. For example, the effects of interspecific aggression on space use has received considerable research attention^10^, especially in terms of range limits and expansion^2,3,11^. In addition to space use, aggression also influences access to foraging resources^12,13^. Although much of this interspecific research addresses interactions on a pairwise scale, such interactions unfold within the context of an entire community of potentially interacting species^14^. Aggressive interactions between multiple species at foraging locations are common^15^, and several studies document the presence of interspecific dominance hierarchies^16,17^. Consequently, the mechanisms explaining the formation and maintenance of such hierarchies may indeed have important resultant effects on community assembly and other ecological processes, yet these mechanisms remain largely unknown.

Dominance and dominance hierarchies within species have been recognized for a considerable amount of time^18–20^. A key explanation for the evolutionary stability of such hierarchies is that they minimize the frequency or intensity of aggression among conspecifics, particularly when the positions in the hierarchy are predictable and familiarity is high among individuals in a group^21^. Indeed, periods of instability or turnover in intraspecific dominance hierarchies are often associated with fitness costs. For instance, aggression was elevated in groups of domestic dogs (*Canis familiaris*) in parts of the dominance hierarchy that were least well-established^22^. Yet, dominance interactions are not limited to members of the same species^16,23–26^. It stands to reason that interspecific dominance hierarchies could be maintained by the same selection pressures that help generate intraspecific hierarchies: individuals evolve to avoid costly aggression, particularly when the outcome of dominance interactions is predictable;^23^ and to do so individuals recognize salient cues^27^. The cues in this case would be cues associated with identifying other species, rather than individuals within the same species^28^.

If species form, recognize, and abide by interspecific dominance hierarchies, one would expect that species pairs that co-occur frequently in space and time should adhere to the structure of the dominance hierarchy better than species pairs that are less familiar with one another. Alternatively, if the outcome of interspecific interactions is too unpredictable, or if the neurological mechanisms that mediate the recognition of rank in a within-species hierarchy are not tuned to heterospecific cues, then dominance hierarchies may instead be maintained as a simple consequence of asymmetries in size or other competitive abilities, with mere despotism generating and maintaining the structure of the hierarchy. If this second hypothesis is true, then the same dominance hierarchy should emerge regardless of how widespread the co-occurrence of members of the hierarchy is. These hypotheses have been difficult to test on a large, interspecific scale due to the intensive observational work required to generate a large interaction database.

To test these hypotheses, we amass and analyze an comparative dataset of interspecific avian interactions from feeders across North America, which adhere to a stable, interspecific dominance hierarchy^16^. We pair each aggressive interaction with multiple ecological, natural history, and social variables based on the species involved in the interaction to examine the factors that shape links in this dominance hierarchy. To dissect the evolutionary drivers of aggressive avian interactions, we generate three distinct variables of aggression that isolate specific aspects of interspecific aggression. These three measures allow us to determine if species pairs abide by an interspecific dominance hierarchy. The first measure is ‘displacement observed’, a simple presence/absence indicator of aggression. The binary displacement observed variable allows us to assess which evolutionary factors generate interspecific aggression. The second variable, deviation from expected encounters (‘DEE’, see below), is a measure of the extent of aggression that adjusts for species abundance. This second variable allows us to determine which factors lead to increased aggression above or below what one would expect if species were interacting at random based on abundance. The third variable, a measure of the directionality of aggression (i.e., the consistency with which individuals of one species ‘win’ in an interaction), allows us to isolate the factors that lead to interspecific dominance within a species pair. If species abide by an interspecific hierarchy, then we would expect species familiarity, in the form of spatial overlap, to lead to a higher likelihood of observing aggression between a pair of species. However, species that abide by an interspecific hierarchy would interact less than expected after controlling for abundance. Similarly, with increasing species familiarity, interactions between species would be resolved towards the dominant member of the pair more than expected.

Here, we show that the degree of species familiarity, as estimated by syntopic overlap, potentially drives the recognition of an interspecific dominance hierarchy. Within the hierarchy, we find that species pairs that show increasing overlap in space and time are more likely to be observed interacting at any point; however, more familiar species pairs interact less than expected after controlling for abundance. Not only do familiar species pairs interact less than expected, aggression between individuals in familiar species pairs more often resolve their aggression towards the relatively dominant species in the pair. Taken together, these effects suggest species recognize their relative position in the interspecific dominance hierarchy.

## Results

### FeederWatch data

There were a total of 88,988 interactions across all species in the full dataset. There were a total of 12,765 unique, co-occurring species pairs in the dataset. From the species pair dataset, we removed interactions with predatory birds and species pairs only found at one feeder location. The resulting data subset had 8,911 unique species pairs; in this subset, displacements were observed between 1,664 unique species pairs, with the sources of aggression consisting of 99 species from 63 genera, and the targets of aggression consisting of 176 species from 97 genera. Interactions were reported from across the United States and southern parts of Canada.

### Likelihood of aggression increases with niche overlap

**Fig. 1:**
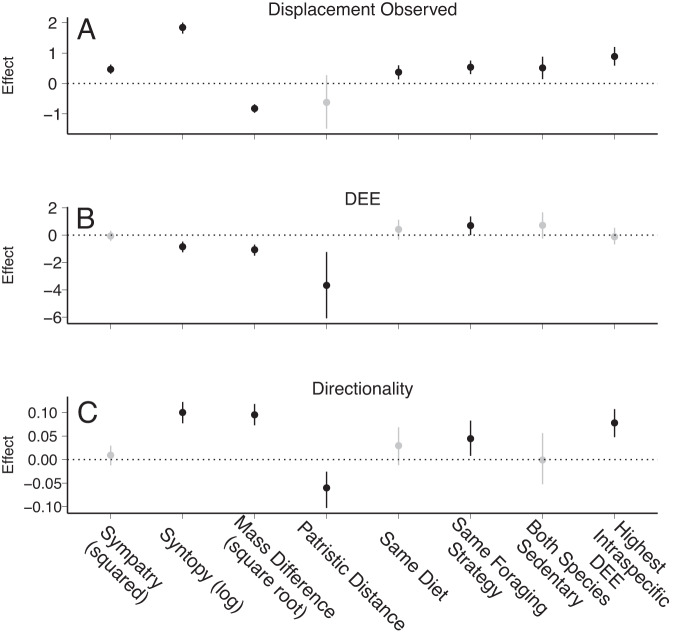
Coefficient estimates for the models predicting variables associated with aggression. Black represents statistically significant effects, whereas gray represents effects not significantly different from zero. Dotted line is a horizontal intercept at 0. Effects generated from model on *n* = 1664 unique species pairs. **A** Coefficients from the phylogenetic logistic mixed model predicting the presence of aggression. **B** Coefficients from the phylogenetic linear mixed model predicting the extent of aggression compared to expected. **C** Coefficients from the phylogenetic linear mixed model predicting the directionality of aggression. Points represent the mean of the fixed effect posterior values and lines represent the 95% credibility intervals. Variables with credibility intervals that do not overlap 0 are depicted in black.

Several variables related to niche overlap predicted the presence of any aggressive displacements between species pairs (Fig. 1; Supplementary Table 1). Species pairs that share a diet (posterior mean = 0.37, 95% CI = 0.14–0.61, *p* = 0.002), have similar foraging ecologies (posterior mean = 0.54, 95% CI = 0.32–0.78, *p* < 0.001), and similar body masses (body mass difference posterior mean = −0.82, 95% CI = −0.97–−0.67, *p* < 0.001) were more likely to be observed in aggressive interactions. We also found that if both species were sedentary (i.e., non-migratory), they were more likely to be observed in an aggressive interaction (posterior mean = −0.50, 95% CI = 0.16–0.88, *p* = 0.006). When one member of a pair of species was generally more aggressive (i.e, had a high intraspecific DEE), the likelihood of observing aggressive interactions was higher (posterior mean = 0.88, 95% CI = 0.57–1.20, *p* < 0.001). Similarly, as species overlap more in range (sympatry posterior mean = 0.47, 95% CI = 0.33–0.61, *p* < 0.001) and specific habitat (syntopy posterior mean = 1.84, 95% CI = 1.66–2.01, *p* < 0.001), they are more likely to be observed in an aggressive displacement.

### Unfamiliarity and body size drive interspecific aggression

**Fig. 2:**
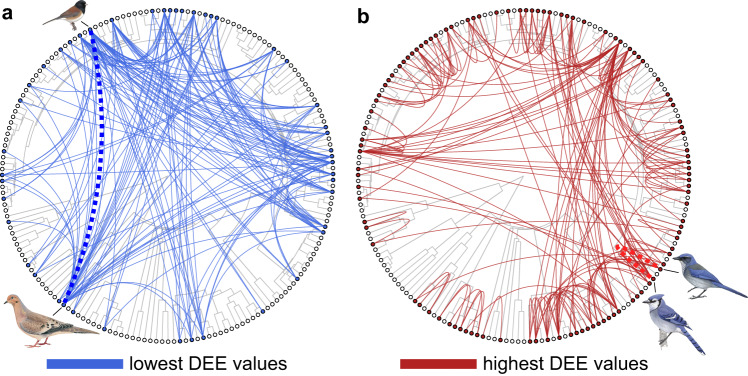
Phylogenetic networks of aggressive interactions at feeders across North America show that species tend to interact most frequently with closest relatives. Plotted are the lowest 10% (**a**) and highest 10% (**b**) of deviation from expected encounters (DEE) values, representing species pairs which interact far less frequently than would be expected if interactions occurred at random and species pairs which interact far more frequently than random expectations, respectively. Dotted connections are representative examples. Example species pair in **A** are a dark-eyed junco (*Junco hyemalis* – illustrated by David Quinn) and mourning dove (*Zenaida macroura* – illustrated by Martin Elliott); example species pair in **B** are a blue jay (*Cyanocitta cristata* – illustrated by Brian Small) and Woodhouse’s scrub jay (*Aphelocoma woodhouseii* – illustrated by Brian Small). Illustrations used with permission, © Birds of the World | Cornell Lab of Ornithology.

In support of the hypothesis that interspecific dominance hierarchies reduce the overall occurrence of costly interspecific aggression between species, we found that species that encounter each other infrequently and are therefore likely unfamiliar with one another engaged in aggressive displacements more frequently than expected by chance—syntopy was negatively associated with DEE (posterior mean = −0.85, 95% CI = −1.24–−0.46, *p* < 0.001; Supplementary Table 2). In these models we accounted for phylogeny. Specifically, patristic distance was negatively associated with DEE in interspecific interactions (posterior mean = −3.62, 95% CI = −6.33–−1.26, *p* = 0.008; Fig. 2); as species become more distantly related, they are less likely to interact based on abundance. Finally, with increasing difference in body size, there was a lower likelihood that species will interact based on abundance (posterior mean = −1.07, 95% CI = −1.46–−0.68, *p* < 0.001).

### Unfamiliarity, body size, and foraging ecology lead to unstructured interactions

**Fig. 3:**
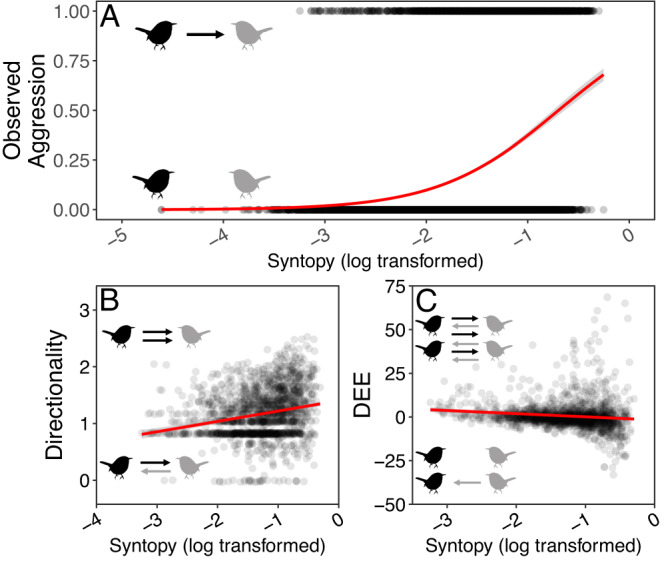
Effects of syntopic range overlap (log transformed) on measures of aggression. Bird figures in each panel represent different species, and arrows represent directed aggression. Black and gray colors represent different hypothetical species. *Thryothorus ludovicianus* silhouette from phylopic.org [CC0 1.0]. **A** Increasing syntopy increases the likelihood of observing interspecific aggression. **B** Increasing syntopy increases directionality. **C** Increasing syntopy decreases DEE. Trendlines are presented from (generalized) linear models, with standard errors depicted in gray.

The increased rate of interaction between unfamiliar species was driven, at least in part, by a breakdown in the interspecific hierarchy at feeders. Species that encounter each other more frequently exhibit more structured interactions—with increasing syntopy, species pairs exhibit higher directionality in aggressive encounters (posterior mean = 0.10, 95% CI = 0.08 – 0.12, *p* < 0.001; Fig. 3); that is, the identities of the winner and the loser were more consistent in these interactions. Yet, other factors impact the structure of interactions at feeders as well (Supplementary Table 3). For instance, species pairs with similar niches exhibit more symmetrical interactions (foraging niche overlap: posterior mean = 0.04, 95% CI = 0.02–0.08, *p* = 0.02; body mass difference: posterior mean = 0.10, 95% CI = 0.07–0.12, *p* < 0.001;). Similarly, species pairs involving a more aggressive species (i.e., those with higher intraspecific DEE values) exhibited higher directionality (posterior mean = 0.08, 95% CI = 0.05–0.11, *p* < 0.001). In these models we account for phylogeny, with more distantly related species exhibited lower directionality (posterior mean = −0.06, 95% CI = −0.10–−0.02, *p* < 0.001). Finally, our analyses lent some support to the observation that the role of mass in determining interaction structure breaks down over evolutionary time—there was an interaction between mass and patristic distance that influenced directionality in the full dataset (*p* < 0.05); though we only recovered a trend in the data subsets (*p* = 0.05 and *p* = 0.06).

## Discussion

Our analyses provide further evidence that aggressive interspecific interactions are more likely to arise between ecologically similar species. We also found support for the hypothesis that familiarity plays a key role in the formation and maintenance of interspecific dominance hierarchies. Species pairs that are unfamiliar with one another engage in more frequent and less structured aggressive interactions at feeders, likely increasing the time and energy spent in such agonistic encounters.

While previous findings have identified mechanisms that reduce aggression within species (e.g. intraspecific dominance hierarchies, dear enemy effects^29^), the results presented here suggest that individuals across many avian species are adjusting their behavior based on heterospecific recognition cues^30^. Recent evidence in reef fish suggests that heterospecifics recognize one another and are more likely to resolve contests with agonistic signaling when resources are relatively abundant^31^. Similarly, analyses of mixed-species groups of parrots at clay licks in the Amazon basin demonstrate that heterospecific recognition influences decisions to join mixed groups, thereby determining species compositions^32^, further underscoring the ability of individuals to respond to complex social tapestries across species boundaries. Our results show that there is a clear benefit to doing so, yet there is likely to be further, as yet unstudied variation in the degree to which species respond to heterospecific cues. Recognizing heterospecifics may be especially valuable for the smaller species in a species pair^33^, for instance, and individuals may quickly learn their relative dominance position compared to familiar species^34^.

In addition to familiarity between species, we also found that niche similarity influenced the likelihood of observing interspecific aggression. First, large differences in body mass were negatively correlated with observing interspecific aggression. Unsurprisingly, species that differed considerably in body size did not enter aggressive contests as readily. That species with large differences in size avoid conflict is in line with previous results^26^, though we did not find an effect of evolutionary distance influencing whether species engaged in contests. However, evolutionary distance was negatively associated with DEE; in other words, more closely related species engage in aggressive contests more often than expected based on abundance. In terms of ecological variables both shared foraging strategy and shared diet led to an increased likelihood of species engaging in aggressive contests. Consequently, species that overlap in resources, in addition to space, tend to have more contests than species with differing foraging ecologies—a result echoed in a previous study on interspecific territoriality during the breeding season^7^. Similarly, species pairs that are both sedentary tend to have a higher likelihood of engaging in aggressive contests over food. However, this result may be due to the structure of the data, as FeederWatch primarily operates during the northern hemisphere winter. Further research into the temporal dynamics of interspecific dominance hierarchies would likely shed additional light on the mechanisms driving their formation and maintenance.

One aspect of the study worth considering is how closely the feeders and associated avian behavior at feeders correspond to interactions over resources in more natural habitats. Specifically, do bird feeders induce increased and different behavioral dynamics than other areas? In some areas, fruiting trees produce concentrated resources that attract a large congregation of birds in both species and abundance^35^. Similarly, feeders may increase aggression above what would be experienced if feeders were not present. However, the aggression and dominance hierarchy present at feeders is a likely representation of these dynamics in other situations. While feeders may represent a relatively novel resource for animals, such concentrated resources are increasing over time. For instance, bird feeding and bird watching are increasing;^36^ additionally, landfills are often areas where larger species congregate^37^. These concentrated resources are increasing with urbanization, and will likely bring more species, including non-native species^38^, into close proximity over time. The facilitation of non-native species at these resources could very well modify the current dominance hierarchies in these areas.

Interspecific dominance at feeders may be modified by other variables in addition to the variables included here. For instance, both intraspecific and interspecific sociality could modify the competitive nature of interactions^14^. Intriguingly, individuals in species that form intraspecific flocks^39^, and fight more amongst conspecifics^40^, tend to be less socially dominant. However, the presence of conspecifics may increase the competitiveness of the group at the expense of competitors^41^.

The combination of findings presented here suggests that individuals may adapt to interspecific hierarchies. These results compliment findings from intraspecific dominance hierarchies^42–44^, suggesting that selection to reduce costly aggression will evolve among familiar individuals both within and among species. Deviations from the patterns presented here might provide fruitful opportunities to understand why certain familiar species engage in more aggression than expected. Specifically, studies on targeted species pairs may illuminate potential traits that produce more constant aggression among familiar individuals^14^. Aggression among species is common among many groups of animals, and given the capacity for heterospecific recognition across many of the same groups of animals, the presence of interspecific dominance hierarchies may be more widespread than traditionally believed.

## Methods

### FeederWatch data

To quantify dominance interactions between species, we employed a large dataset of interspecific displacements at feeders derived from data from Project FeederWatch. Project FeederWatch is a program run by the Cornell Laboratory of Ornithology where participants can submit observed interactions between birds at bird feeders^45^. Specifically, observers submitted information about displacements, identifying the species of the winner and loser of each successful displacement. We incorporate interaction data from 2015-2020 with submitted interactions from across North America.

### Database

In calculating certain variables (e.g. DEE, see below), we found that some rare species pairs yielded extreme values. These extreme values sometimes had high leverage in the models, and to generate a conservative dataset and subsequent set of analyses, we subset the data in multiple ways for the analysis described below. Specifically, we removed species pairs that were found at only one single feeder location; several of these singleton pairs had high DEE values and we removed them to eliminate the effects of their high statistical leverage. We also removed any species pairs that include a predatory bird (e.g. a hawk or falcon). Since predatory birds can be observed around feeders they are in the overall dataset, but are removed from the focal subset of data. We ran all analyses on the full dataset, the dataset with species pairs observed at more than one feeder, and a focal dataset removing both predatory birds and species pairs observed at more than one feeder. We report the results of the most conservative analysis (i.e. the analysis with both predators and singleton pairs removed) here and the results of the other datasets in the supplementary information (Supplementary Tables 4–9). Importantly, the main results of all three sets of analyses were qualitatively identical.

### Aggression variables

From this dataset, we calculated several variables to characterize the network of agonistic interactions between species. First, to identify factors that predict which species pairs exhibited interspecific aggression, and therefore contribute to the formation of interspecific dominance hierarchies, we created a simple binary variable to indicate whether two species were ever observed in a displacement, for which we restricted the data to species that ever co-occurred at any feeder and therefore could have conceivably been observed interacting.

Next, to test the key prediction of the alternative hypotheses for the maintenance of interspecific dominance hierarchies, we used two indices to characterize the adherence to the dominance hierarchy. The first—a standardized effect size (SES) value following refs. ^28,46^—is an index of the deviation of the frequency of observed displacements from the number expected if species interact at random. To improve clarity, we now refer to this SES as ‘Deviation from Expected Encounters’, or DEE. We used abundance data from FeederWatch counts to simulate interaction datasets where, for each feeder, we simulated the same number of displacements as were recorded in the observed dataset, sampling two interacting individuals in proportion to their abundances at the observed feeder. We then aggregated simulated fights across all feeders in the empirical dataset, creating a matrix of simulated displacements. We repeated this 10,000 times and calculated the mean and standard deviation of the number of simulated fights across these 10,000 simulated matrices. DEE values were then calculated by dividing the difference between the observed number of pairwise interactions and the mean expected number of such interactions by the standard deviation of the simulated number of interactions. Consequently, positive values of DEE represent more interactions than expected based on abundances, whereas negative values of DEE represent fewer interactions than expected based on abundances. Notably, while each species pair has an interspecific DEE value, each species has its own, intraspecific DEE value, as well (generated from expected extent of aggression towards conspecifics). Secondly, we calculated an index of directionality to capture the asymmetry in aggressive interactions in each species pair using the index developed by ref. ^26^

1 $$\sqrt{{{{{\mathrm{ln}}}}}(({{{{{{\rm{N}}}}}}}_{{{{{{\rm{wins}}}}}}\,{{{{{\rm{by}}}}}}\,{{{{{\rm{dominant}}}}}}}+1)/({{{{{{\rm{N}}}}}}}_{{{{{{\rm{wins}}}}}}\,{{{{{\rm{by}}}}}}\,{{{{{\rm{subordinate}}}}}}}+1))}$$

where *N*_wins by dominant_ is the number of displacements won by the species in the pair which won the highest number of displacements (i.e., the dominant species), and *N*_wins by subordinate_ is the number of displacements won by the species in the pair which won the fewest number of displacements (i.e., the subordinate species). Zero values, therefore, indicate that members of both species displace individuals of the other species at equal rates (i.e., that interspecific aggression is symmetrical), while high values correspond to asymmetric cases where the dominant species primarily displaces the subordinate species.

### Trait data

To identify factors that predict the occurrence of interspecific aggression, we collected data on numerous morphological and ecological traits. Data on species body mass, diet, foraging behavior, and migration strategy were obtained from ref. ^47^. To test whether species with similar niches are more likely to engage in interspecific aggression at feeders, we generated several variables using the trait data for species pairs. Specifically, we determined whether species pairs shared the same general diet, the same foraging strategy, the same migratory status, and whether both species were passerines or not, coding these as binomial variables (i.e., yes = 1, no = 0).

### Sympatry and syntopy indices from eBird

To test whether the magnitude of aggressive interactions in dominance hierarchies was influenced by species familiarity with one another, we calculated two estimates of spatial overlap for each species pair. Estimates of species pair range overlap (i.e., sympatry) and habitat overlap (i.e., syntopy) were produced from data collected from eBird.org^48^, following the approach developed in ref. ^7^ To this end, data from eBird were downloaded for each species in our analyses, restricting data to complete checklists with observations collected in the United States or Canada between the months of November to March (the months in which the FeederWatch project runs) for the five nonbreeding seasons between 2015 and 2020. These data were further filtered to only include lists collected by observers who traveled less than 0.25 miles to ensure that all birds in each checklist occurred in similar areas.

We used these filtered eBird observations to calculate an index of non-breeding range overlap (sympatry) for each species pair following ref. ^7^. Briefly, this index is similar to the commonly used Szymkiewicz-Simpson overlap index and was calculated using the formula:

2 $$\left\{\begin{array}{c}\frac{1}{{{{{{\rm{Y}}}}}}}\mathop{\sum }\limits_{{{{{{\rm{i}}}}}}=1}^{{{{{{\rm{Y}}}}}}}\frac{{{{{{{\rm{N}}}}}}1,2}_{{{{{{\rm{i}}}}}}}}{{{{{{{\rm{N}}}}}}1}_{{{{{{\rm{i}}}}}}}},\, {{{{{\rm{if}}}}}}\,{{{{{{\rm{N}}}}}}1}_{{{{{{\rm{i}}}}}}} \, < \,{{{{{{\rm{N}}}}}}2}_{{{{{{\rm{i}}}}}}}\\ \frac{1}{{{{{{\rm{Y}}}}}}}\mathop{\sum }\limits_{{{{{{\rm{i}}}}}}=1}^{{{{{{\rm{Y}}}}}}}\frac{{{{{{{\rm{N}}}}}}2,1}_{{{{{{\rm{i}}}}}}}}{{{{{{{\rm{N}}}}}}2}_{{{{{{\rm{i}}}}}}}},\, {{{{{\rm{if}}}}}}{{{{{{\rm{N}}}}}}2}_{{{{{{\rm{i}}}}}}} \, < \, {{{{{{\rm{N}}}}}}1}_{{{{{{\rm{i}}}}}}}\end{array}\right.$$

where *N*1,2_*i*_ refers to the number of unique locations where species 1 was observed in sympatry with (within 24.5 miles of) species 2 in year I, *N*1_*i*_ refers to the number of unique locations where species 1 was observed in year *i*, and *Y* refers to the total number of years (5, in this case).

Similarly, we calculated an index of fine-scale non-breeding habitat overlap (‘syntopy’) for each species pair using the filtered eBird observations, calculated using the formula:

3 $$\frac{1}{{{{{{\rm{Y}}}}}}}\mathop{\sum }\limits_{{{{{{\rm{i}}}}}}=1}^{{{{{{\rm{Y}}}}}}}\frac{({{{{{{\rm{n}}}}}}1,2}_{{{{{{\rm{i}}}}}}}+{{{{{{\rm{n}}}}}}2,1}_{{{{{{\rm{i}}}}}}})}{({{{{{{\rm{N}}}}}}1,2}_{{{{{{\rm{i}}}}}}}+{{{{{{\rm{N}}}}}}2,1}_{{{{{{\rm{i}}}}}}})}$$

where *n*1,2_i_ refers to the number of unique locations where species 1 was observed in syntopy with (within 0.25 miles of) species 2 in year i, and other terms are as above (as in ref. ^7^). Species pairs with high syntopy values, therefore, are more likely to be found in close proximity and, crucially, be familiar with each other.

### Phylogeny

To account for the non-independence among species resulting from their shared evolutionary history, we conducted analyses that incorporate the avian phylogeny. We used a maximum clade credibility tree constructed from the posterior distribution of trees available on birdtree.org^49^, using the taxonomic backbone from Hackett et al.^50^. To this phylogeny, we added recent splits (winter wren [*T. hiemalis*] and Pacific wren [*T. pacificus*]; California scrub jay [*Aphelocoma californica*] and Woodhouse’s scrub jay [*A. woodhouseii*]) following published estimates for the timing of these splits^51,52^ (Supplementary Data 1). We then used the R package ape^53^ to calculate patristic distance for each species pair (the branch length separating them; i.e., twice the time separating each species in the pair from their most recent common ancestor) from this phylogeny.

### Statistical analyses

All analyses were conducted using R v. 4.1.2 (). Given that each observation in our dataset is derived from a species pair, we used phylogenetic linear mixed models (PLMMs) adapted for species interaction data^54,55^ to identify which factors predict the occurrence, strength, and direction of interactions observed by FeederWatch participants. Each model was fit using the R package MCMCglmm^56^, including species identity and the phylogeny as random effects, specifying which node in the phylogeny represent the most recent common ancestor linking each species pair. We used an uninformative, inverse Wishart distribution as a prior distribution for the random effects, fixing the residual variance at 1 for binomial models. To fit the models, we ran MCMC chains for 5 ×10^5^ generations, logging the output every 500 generations and ignoring the first 1,000 generations as burn-in. For each model we used uninformative inverse-gamma priors. We fit each model four times, verified convergence by visually inspecting trace plots and confirming that values Gelman-Rubin values were close to 1 (1-1.01) in the R-package coda^57,58^, and then merged the converged chains. From these PLMMs, we calculated the phylogenetic signal, λ, from the random effects as the phylogenetic intraclass correlation^59^. All statistical tests were two-sided tests.

To test the hypothesis that the effect of body size on the directionality of interactions varies as a function of evolutionary relatedness, we added an interaction term between the body mass difference and patristic distance for the PLMM fit to the directionality dataset. When analyzing the unexplained extent of aggressive interactions (DEE) between species and directionality, we subset the dataset to only those species pairs where aggressive interactions were observed.

### Reporting summary

Further information on research design is available in the Nature Portfolio Reporting Summary linked to this article.

### Supplementary information

Supplementary Information

Peer Review File

Description of Additional Supplementary Files

Supplementary Data 1

Reporting Summary

### Source data

Source Data

## Data Availability

The species pair data generated in this study can be found in the figshare repository DOI: 10.6084/m9.figshare.24309697. The data are under no restriction and can be accessed freely. Raw individual interaction data are provided as a Source Data file. Source data are provided with this paper.

## Code availability

Code is available in the figshare repository DOI: 10.6084/m9.figshare.24309697.
