# Supplementary material for: Unfamiliarity generates costly aggression in interspecific avian dominance hierarchies
Source: Nat Commun. 2024 Jan 6;15:335. doi: 10.1038/s41467-023-44613-0 (PMC10771497; doi:10.1038/s41467-023-44613-0)
Supplement: Supplementary file 1 — Supplementary Information [file 41467_2023_44613_MOESM1_ESM.pdf]

Supplementary Information for Leighton et al.

Supplementary Figures

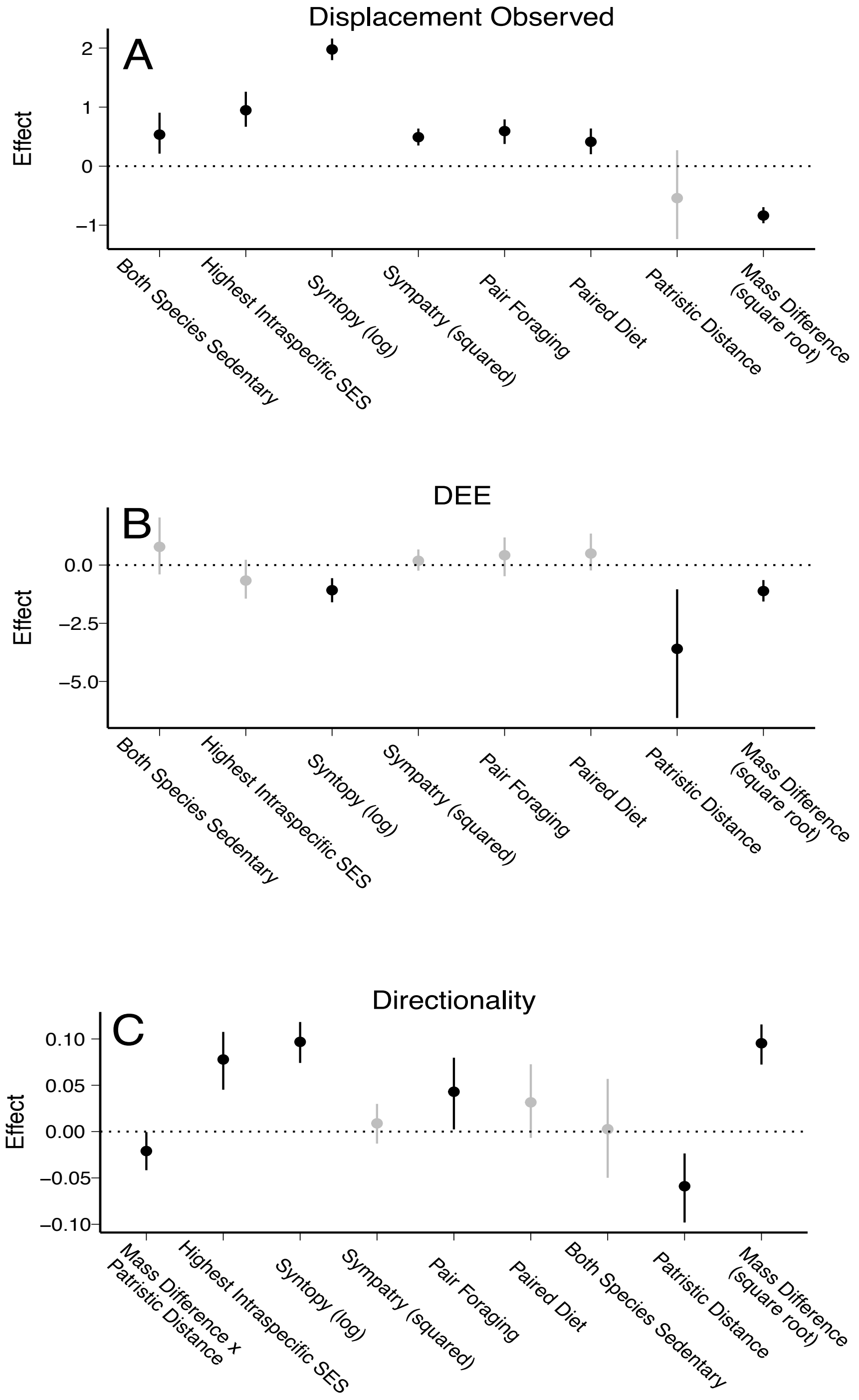

Supplemental Figure 1: Coefficient estimates for the models predicting variables associated with aggression in full dataset. (A) Coefficients from the phylogenetic linear mixed model predicting presence of aggression. (B) Coefficients from the phylogenetic linear mixed model predicting the extent of aggression compared to expected. (C) Coefficients from the phylogenetic logistic mixed model predicting the directionality of aggression. Points represent fixed effect posterior values and lines represent the 95% credibility intervals.

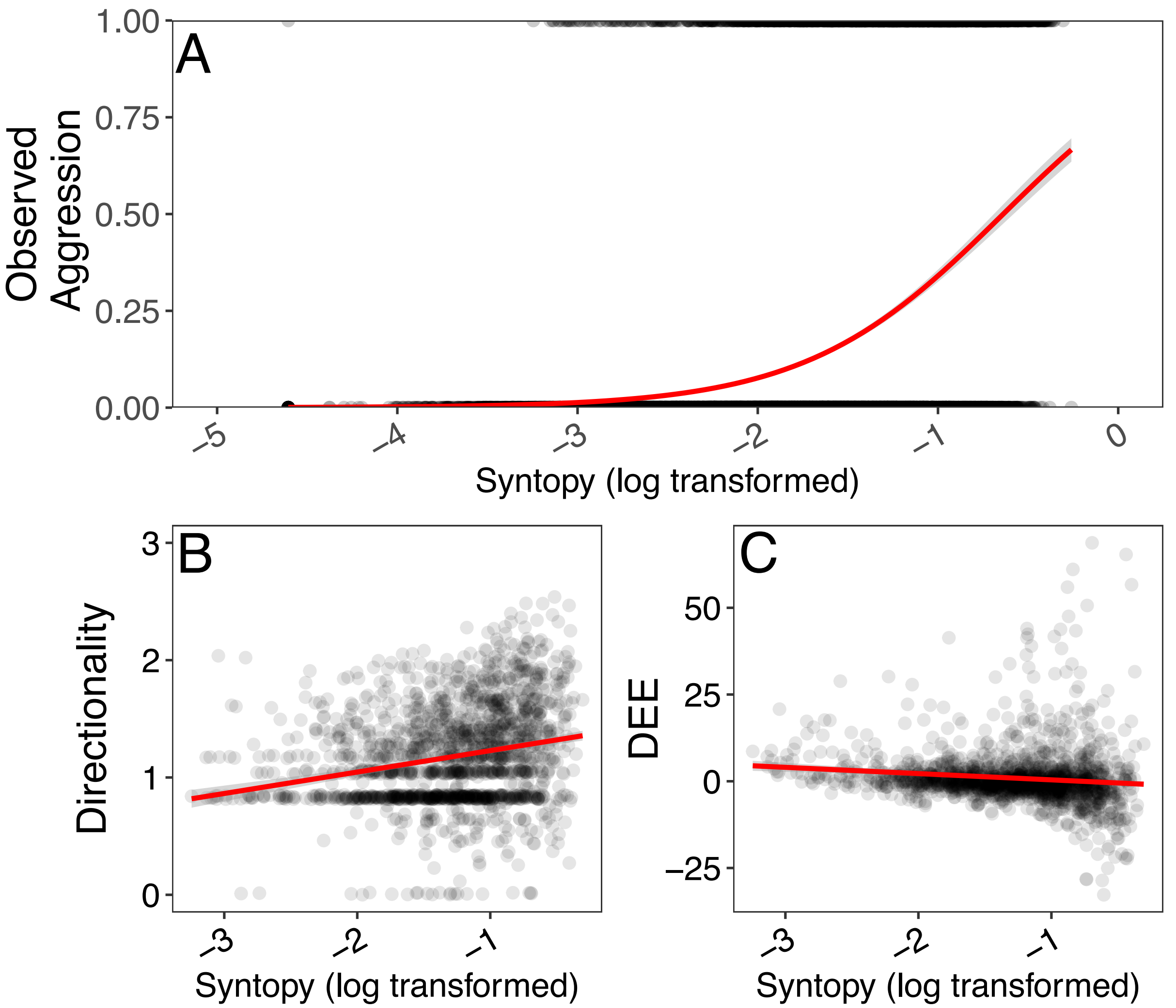

Supplemental Figure 2: Effects of syntopic range overlap (log transformed) on measures of aggression in full dataset. (A) Increasing syntopy increases the likelihood of observing interspecific aggression. (B) Increasing syntopy increases directionality. (C) Increasing syntopy decreases DEE. All lines are model prediction lines with glm smooth.

Supplementary Information for Leighton et al.

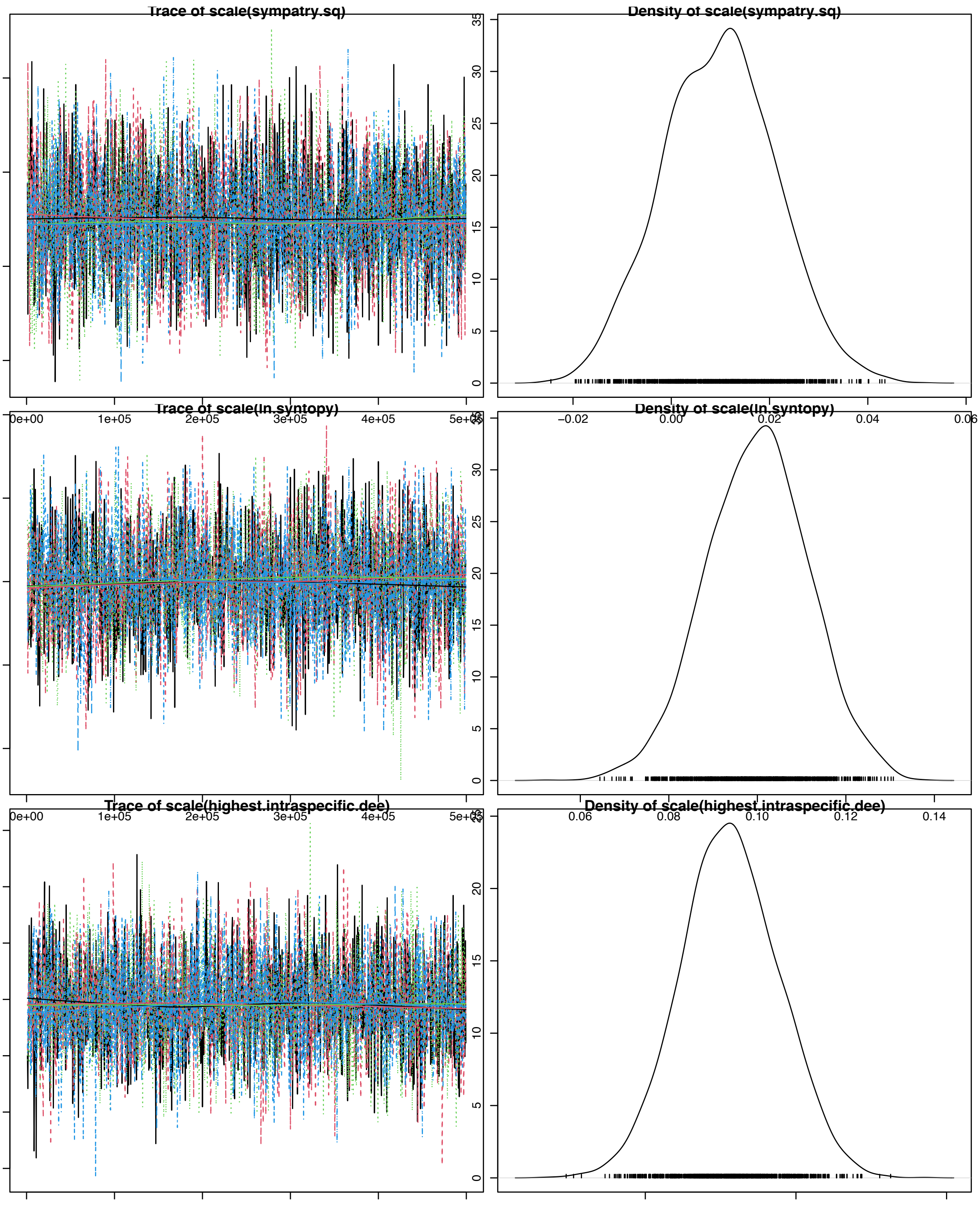

Supplementary Information for Leighton et al.

Supplemental Figure 3: Overlayed traces of MCMC chains for model analyzing directionality. All variable traces are qualitatively equal to the variables featured here.

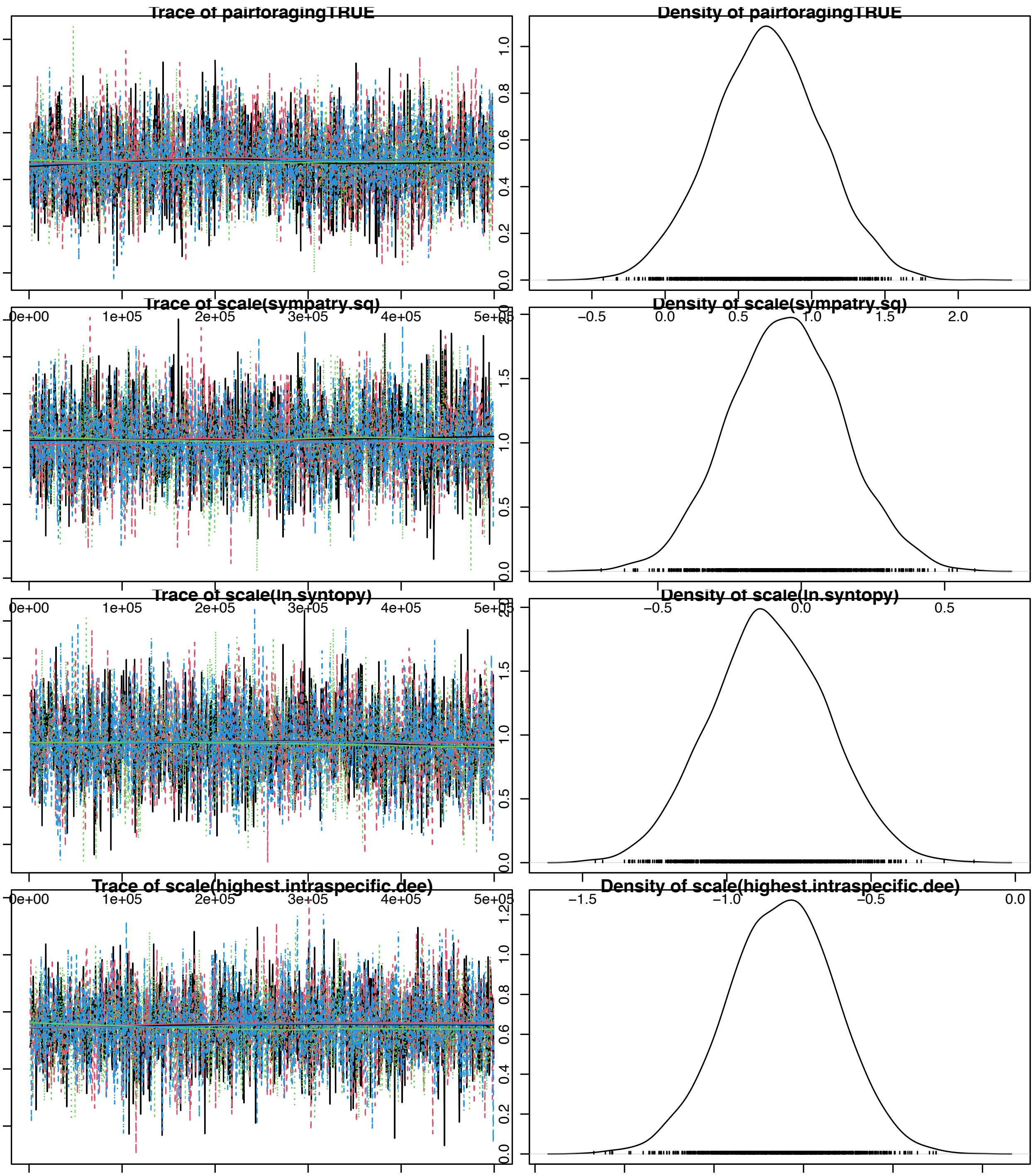

Supplemental Figure 4: Overlayed traces of MCMC chains for model analyzing DEE. All variable traces are qualitatively equal to the variables featured here.

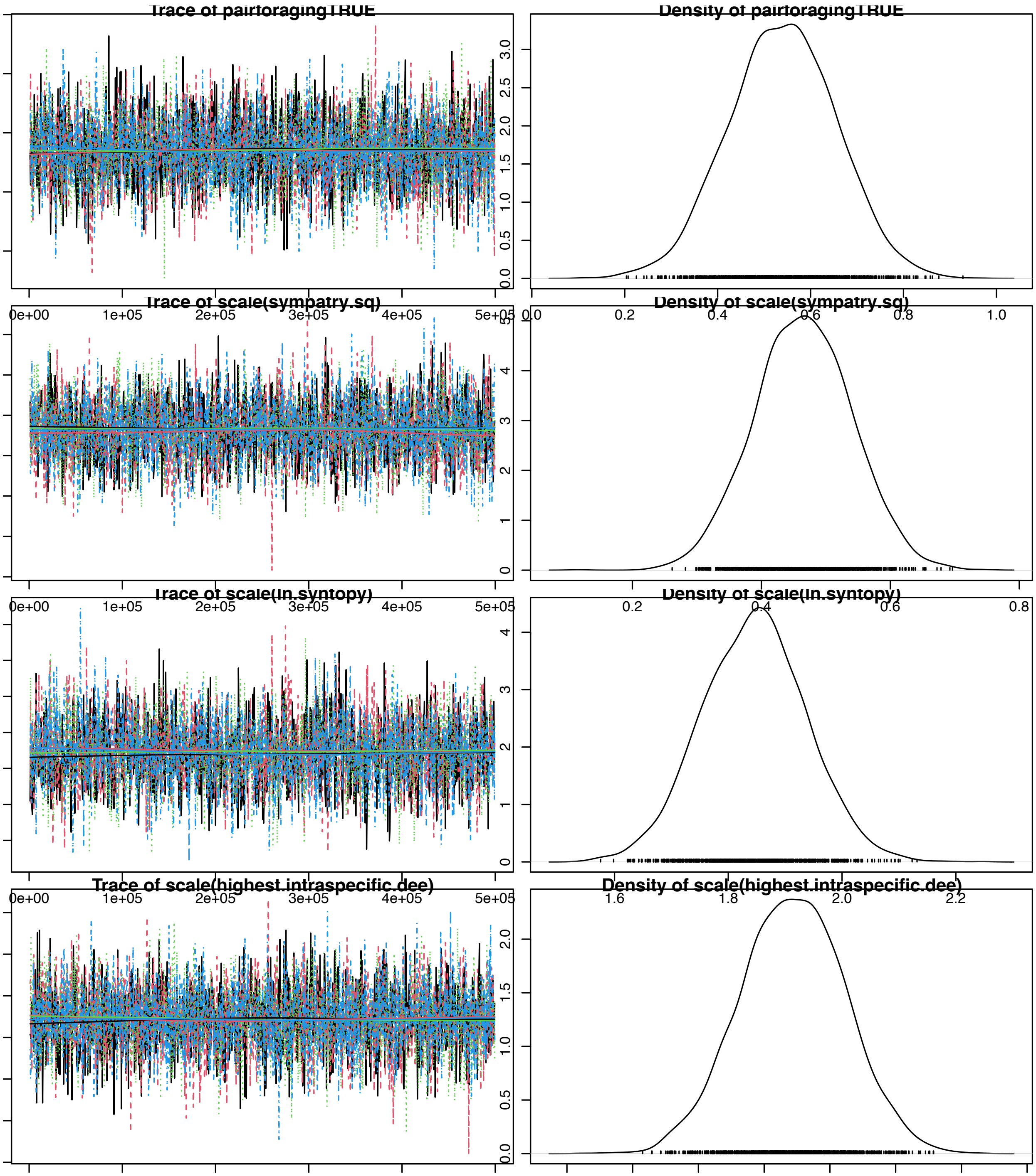

Supplemental Figure 5: Overlayed traces of MCMC chains for model analyzing whether an interaction was observed. All variable traces are qualitatively equal to the variables featured here.

Supplementary Information for Leighton et al.

Supplementary Tables

Supplemental Table 1: Analysis of conservative dataset without predators and with species pairs observed at more than one location. Variables in full model predicting whether aggressive interspecific interactions were observed (presence). If the variable was transformed that is included in parentheses following the variable. The Gelman-Rubin diagnostic was 1.00 or 1.01 for all variables in this model. The phylogenetic signal for the presence of aggressive interactions was 0.694 (95% credibility interval: 0.689 – 0.700). The model was a Bayesian phylogenetic logistic regression, that employed a two-sided test of significance with pMCMC values. Each effect is compared to whether it is significantly different than zero.

	Posterior mean	Lower-95% CI	Upper-95% CI	Effective Sample	pMCMC
Intercept	-2.86	-5.86	0.22	998.00	0.064
Body Mass Difference (square root)	-0.82	-0.97	-0.67	905.70	< 0.001
Patristic Distance	-0.61	-1.48	0.26	998.00	0.174
Pair Diet: True	0.37	0.14	0.61	998.00	0.002
Pair Foraging: True	0.54	0.32	0.78	1248.80	< 0.001
Sympatric Range Overlap (square root)	0.47	0.33	0.61	793.10	< 0.001
Syntopic Range Overlap (log)	1.84	1.66	2.01	674.40	< 0.001
Highest Intraspecific DEE	0.89	0.57	1.20	998.00	< 0.001
Both Sedentary: True	0.50	0.16	0.88	998.00	0.006

Supplemental Table 2: Analysis of conservative dataset without predators and with species pairs observed at more than one location. Variables in full model predicting the deviations from expected extent (DEE) of aggressive interspecific interactions. If the variable was transformed that is included in parentheses following the variable. The Gelman-Rubin diagnostic was 1.00 for all variables except for one, and the remaining variable was 1.01, in this model. The phylogenetic signal for the extent of aggressive interactions was 0.747 (95% credibility interval: 0.742 – 0.752). The model was a Bayesian phylogenetic regression, that employed a two-sided test of significance with pMCMC values. Each effect is compared to whether it is significantly different than zero.

	Posterior mean	Lower-95% CI	Upper-95% CI	Effective Sample	pMCMC
Intercept	6.92	-5.33	18.18	998.00	0.234
Body Mass Difference (square root)	-1.07	-1.46	-0.69	1135.90	< 0.001
Patristic Distance	-3.62	-6.33	-1.26	998.00	0.008
Paired Diet: True	0.45	-0.27	1.14	998.00	0.222
Pair Foraging: True	0.69	-0.11	1.37	998.00	0.080
Sympatric Range Overlap (square root)	-0.05	-0.46	0.33	892.10	0.817
Syntopic Range Overlap (log)	-0.85	-1.24	-0.46	858.50	< 0.001
Highest Intraspecific DEE	-0.12	-0.68	0.48	998.00	0.697
Both Sedentary: True	0.70	-0.37	1.66	998.00	0.172

Supplementary Information for Leighton et al.

Supplemental Table 3: Analysis of conservative dataset without predators and with species pairs observed at more than one location. Variables in full model predicting directionality of aggressive interspecific interactions. If the variable was transformed that is included in parentheses following the variable. The Gelman-Rubin diagnostic was 1.01 for all variables in this model. The phylogenetic signal for directionality was 0.063 (95% credibility interval: 0.058 – 0.068). The model was a Bayesian phylogenetic regression, that employed a two-sided test of significance with pMCMC values. Each effect is compared to whether it is significantly different than zero.

	Posterior mean	Lower-95% CI	Upper-95% CI	Effective Sample	pMCMC
Intercept	1.10	0.97	1.24	998.00	<0.001
Body Mass Difference (square root)	0.10	0.07	0.12	998.00	<0.001
Patristic Distance	-0.06	-0.10	-0.02	988.90	0.02
Both Sedentary: True	0.00	-0.06	0.05	998.00	0.915
Paired Diet: True	0.03	-0.01	0.07	998.00	0.184
Paired Foraging: True	0.04	0.00	0.08	807.20	0.018
Sympatric Range Overlap (square root)	0.01	-0.01	0.03	998.00	0.366
Syntopic Range Overlap (log)	0.10	0.08	0.12	888.40	<0.001
Highest Intraspecific DEE	0.08	0.05	0.11	873.20	<0.001
Body Mass Difference x Patristic Distance	-0.02	-0.04	0.00	998.00	0.052

Supplemental Table 4: Analysis of the full dataset. Variables in full model predicting whether aggressive interspecific interactions were observed (presence) using full dataset. If the variable was transformed that is included in parentheses following the variable. The Gelman-Rubin diagnostic was 1.00 or 1.01 for all variables in this model. The phylogenetic signal for the presence of aggressive interactions was 0.54 (95% confidence interval: 0.28 – 0.73). The model was a Bayesian phylogenetic logistic regression, that employed a two-sided test of significance with pMCMC values. Each effect is compared to whether it is significantly different than zero.

	Posterior mean	Lower-95% CI	Upper-95% CI	Effective Sample	pMCMC
Intercept	-4.097	-6.943	-1.013	896.025	0.015
Body Mass Difference (square root)	-0.835	-0.974	-0.693	822.225	<0.001
Patristic Distance	-0.546	-1.261	0.210	1003.050	0.146
Pair Diet: True	0.411	0.198	0.634	1016.800	0.002
Pair Foraging: True	0.589	0.371	0.799	1034.250	<0.001
Sympatric Range Overlap (square root)	0.491	0.346	0.630	901.900	<0.001
Syntopic Range Overlap (log)	1.977	1.793	2.153	683.050	<0.001
Highest Intraspecific DEE	0.938	0.643	1.230	1024.550	<0.001
Both Sedentary: True	0.533	0.197	0.911	1034.525	0.006

Supplemental Table 5: Analysis of the full dataset. Variables in full model predicting the deviations from expected extent (DEE) of aggressive interspecific interactions in full dataset. If the variable was transformed that is included in parentheses following the variable. The Gelman-Rubin diagnostic was 1.00 for all variables except for one, and the remaining variable was 1.01, in this model. The phylogenetic signal for the extent of aggressive interactions was 0.41 (95% confidence interval: 0.21 – 0.62). The model was a Bayesian phylogenetic regression, that employed a two-sided test of significance with pMCMC values. Each effect is compared to whether it is significantly different than zero.

	Posterior mean	Lower-95% CI	Upper-95% CI	Effective Sample	pMCMC
Intercept	7.596	-3.265	18.701	1211.000	0.172
Body Mass Difference (square root)	-1.176	-1.555	-0.741	1094.000	<0.001
Patristic Distance	-3.737	-6.094	-1.569	998.000	0.002
Paired Diet: True	0.344	-0.328	1.143	998.000	0.355
Pair Foraging: True	0.637	-0.024	1.466	998.000	0.082
Sympatric Range Overlap (square root)	0.006	-0.426	0.392	1101.000	0.988
Syntopic Range Overlap (log)	-1.082	-1.505	-0.624	998.000	<0.001
Highest Intraspecific DEE	-0.340	-0.964	0.383	998.000	0.371
Both Sedentary: True	1.078	0.109	2.236	998.000	0.048

Supplemental Table 6: Analysis of the full dataset. Variables in full model predicting directionality of aggressive interspecific interactions in full dataset. If the variable was transformed that is included in parentheses following the variable. The Gelman-Rubin diagnostic was 1.00 for all variables in this model. The phylogenetic signal for directionality was 0.06 (95% confidence interval: 0.00 – 0.25). The model was a Bayesian phylogenetic regression, that employed a two-sided test of significance with pMCMC values. Each effect is compared to whether it is significantly different than zero.

	Posterior mean	Lower-95% CI	Upper-95% CI	Effective Sample	pMCMC
Intercept	1.099	0.980	1.222	998.000	< 0.001
Body Mass Difference (square root)	0.095	0.074	0.115	998.000	< 0.001
Patristic Distance	-0.058	-0.091	-0.020	1297.400	0.006
Both Sedentary: True	-0.001	-0.058	0.055	998.000	0.976
Paired Diet: True	0.033	-0.009	0.070	998.000	0.134
Paired Foraging: True	0.042	0.000	0.079	1095.800	0.046
Sympatric Range Overlap (square root)	0.010	-0.011	0.032	703.300	0.385
Syntopic Range Overlap (log)	0.100	0.079	0.124	998.000	< 0.001
Highest Intraspecific DEE	0.077	0.046	0.106	1570.100	< 0.001
Body Mass Difference x Patristic Distance	-0.021	-0.041	-0.001	998.000	0.044

Supplemental Table 7: Analysis of the dataset with species pairs observed at more than one feeder location. Variables in full model predicting whether aggressive interspecific interactions were observed (presence) using full dataset. If the variable was transformed that is included in parentheses following the variable. The Gelman-Rubin diagnostic was 1.00 or 1.01 for all variables in this model. The phylogenetic signal for the presence of aggressive interactions was 0.54 (95% confidence interval: 0.28 – 0.73). The model was a Bayesian phylogenetic logistic regression, that employed a two-sided test of significance with pMCMC values. Each effect is compared to whether it is significantly different than zero.

	Posterior mean	Lower-95% CI	Upper-95% CI	Effective Sample	pMCMC
Intercept	-3.14	-6.29	-0.23	998.00	0.05411
Body Mass Difference (square root)	-0.85	-0.99	-0.68	836.80	< 0.001
Patristic Distance	-0.57	-1.43	0.24	768.60	0.15832
Pair Diet: True	0.41	0.15	0.64	998.00	< 0.001
Pair Foraging: True	0.57	0.36	0.80	998.00	< 0.001
Sympatric Range Overlap (square root)	0.46	0.33	0.61	998.00	< 0.001
Syntopic Range Overlap (log)	1.81	1.64	1.97	767.60	< 0.001
Highest Intraspecific DEE	0.92	0.60	1.23	998.00	< 0.001
Both Sedentary: True	0.56	0.24	0.96	837.10	0.00401

Supplemental Table 8: Analysis of the dataset with species pairs observed at more than one feeder location. Variables in full model predicting the deviations from expected extent (DEE) of aggressive interspecific interactions in full dataset. If the variable was transformed that is included in parentheses following the variable. The Gelman-Rubin diagnostic was 1.00 for all variables except for one, and the remaining variable was 1.01, in this model. The phylogenetic signal for the extent of aggressive interactions was 0.41 (95% confidence interval: 0.21 – 0.62). The model was a Bayesian phylogenetic regression, that employed a two-sided test of significance with pMCMC values. Each effect is compared to whether it is significantly different than zero.

	Posterior mean	Lower-95% CI	Upper-95% CI	Effective Sample	pMCMC
Intercept	6.31	-7.52	20.83	998.00	0.385
Body Mass Difference (square root)	-1.06	-1.48	-0.66	998.00	<0.001
Patristic Distance	-3.41	-5.84	-0.22	998.00	0.018
Paired Diet: True	0.51	-0.23	1.27	998.00	0.188
Pair Foraging: True	0.64	-0.11	1.36	998.00	0.104
Sympatric Range Overlap (square root)	-0.04	-0.42	0.39	998.00	0.856
Syntopic Range Overlap (log)	-0.81	-1.21	-0.38	998.00	<0.001
Highest Intraspecific DEE	-0.14	-0.75	0.46	998.00	0.663
Both Sedentary: True	0.71	-0.22	1.75	998.00	0.16

Supplemental Table 9: Analysis of the dataset with species pairs observed at more than one feeder location. Variables in full model predicting directionality of aggressive interspecific interactions in full dataset. If the variable was transformed that is included in parentheses following the variable. The Gelman-Rubin diagnostic was 1.00 for all variables in this model. The phylogenetic signal for directionality was 0.06 (95% confidence interval: 0.00 – 0.25). The model was a Bayesian phylogenetic regression, that employed a two-sided test of significance with pMCMC values. Each effect is compared to whether it is significantly different than zero.

	Posterior mean	Lower-95% CI	Upper-95% CI	Effective Sample	pMCMC
Intercept	1.10	0.96	1.22	1086.00	<0.001
Body Mass Difference (square root)	0.10	0.07	0.12	998.00	<0.001
Patristic Distance	-0.06	-0.10	-0.02	998.00	0.024
Both Sedentary: True	0.00	-0.06	0.05	998.00	0.9559
Paired Diet: True	0.03	-0.01	0.07	998.00	0.1503
Paired Foraging: True	0.04	0.01	0.09	998.00	0.0441
Sympatric Range Overlap (square root)	0.01	-0.01	0.03	998.00	0.4489
Syntopic Range Overlap (log)	0.10	0.08	0.12	998.00	<0.001
Highest Intraspecific DEE	0.08	0.05	0.11	998.00	<0.001
Body Mass Difference x Patristic Distance	-0.02	-0.04	0.00	998.00	0.0581
