# Supplementary material for: Unfamiliarity generates costly aggression in interspecific avian dominance hierarchies
Source: Nat Commun. 2024 Jan 6;15:335. doi: 10.1038/s41467-023-44613-0 (PMC10771497; doi:10.1038/s41467-023-44613-0)
Supplement: Supplementary file 3 — Description of Additional Supplementary Files [file 41467_2023_44613_MOESM3_ESM.pdf]

Description of Additional Supplementary Files

File Name: Supplementary Data 1

Description: Phylogenetic tree used in analysis
