# Supplementary material for: Unfamiliarity generates costly aggression in interspecific avian dominance hierarchies
Source: Nat Commun. 2024 Jan 6;15:335. doi: 10.1038/s41467-023-44613-0 (PMC10771497; doi:10.1038/s41467-023-44613-0)
Supplement: Supplementary file 5 — Reporting Summary [file 41467_2023_44613_MOESM5_ESM.pdf]

nature portfolio

Corresponding author(s): Gavin Leighton

Last updated by author(s): Nov 17, 2023

Reporting Summary

Nature Portfolio wishes to improve the reproducibility of the work that we publish. This form provides structure for consistency and transparency in reporting. For further information on Nature Portfolio policies, see our Editorial Policies and the Editorial Policy Checklist.

Statistics

For all statistical analyses, confirm that the following items are present in the figure legend, table legend, main text, or Methods section.

n/a	Confirmed	
☐	☒	The exact sample size (*n*) for each experimental group/condition, given as a discrete number and unit of measurement
☐	☒	A statement on whether measurements were taken from distinct samples or whether the same sample was measured repeatedly
☐	☒	The statistical test(s) used AND whether they are one- or two-sided<br>*Only common tests should be described solely by name; describe more complex techniques in the Methods section.*
☐	☒	A description of all covariates tested
☐	☒	A description of any assumptions or corrections, such as tests of normality and adjustment for multiple comparisons
☐	☒	A full description of the statistical parameters including central tendency (e.g. means) or other basic estimates (e.g. regression coefficient) AND variation (e.g. standard deviation) or associated estimates of uncertainty (e.g. confidence intervals)
☐	☒	For null hypothesis testing, the test statistic (e.g. *F*, *t*, *r*) with confidence intervals, effect sizes, degrees of freedom and *P* value noted<br>*Give P values as exact values whenever suitable.*
☐	☒	For Bayesian analysis, information on the choice of priors and Markov chain Monte Carlo settings
☐	☒	For hierarchical and complex designs, identification of the appropriate level for tests and full reporting of outcomes
☐	☒	Estimates of effect sizes (e.g. Cohen's *d*, Pearson's *r*), indicating how they were calculated

Our web collection on statistics for biologists contains articles on many of the points above.

Software and code

Policy information about availability of computer code

Data collection	Bird counts were submitted by participants via the FeederWatch data entry portal (https://feederwatch.org/) and, beginning in 2019, via the app available for both iPhone and Android. In 2016, interaction data were added via a standalone online platform that linked with FeederWatch. From 2017 onwards, the interactions were added directly via the FeederWatch portal.
Data analysis	All analyses were run in the R computing environment (R v. 4.1.2) using the packages ape (v. 5.0) and MCMCglmm (v. 2.35). Plots were generated with ggplot2 (v. 3.4.3), patchwork (v 1.1.3), and circlize (v. 0.4.15). An example script for fitting PLMMs is available on figshare: https://doi.org/10.6084/m9.figshare.24309697.v1

For manuscripts utilizing custom algorithms or software that are central to the research but not yet described in published literature, software must be made available to editors and reviewers. We strongly encourage code deposition in a community repository (e.g. GitHub). See the Nature Portfolio guidelines for submitting code & software for further information.

Data

Policy information about availability of data

All manuscripts must include a data availability statement. This statement should provide the following information, where applicable:

- Accession codes, unique identifiers, or web links for publicly available datasets
- A description of any restrictions on data availability
- For clinical datasets or third party data, please ensure that the statement adheres to our policy

All data (species level data, species pair data, and the phylogeny) used in analyses are available on figshare: https://doi.org/10.6084/m9.figshare.24309697.v1

Research involving human participants, their data, or biological material

Policy information about studies with human participants or human data. See also policy information about sex, gender (identity/presentation), and sexual orientation and race, ethnicity and racism.

Reporting on sex and gender	N/A
Reporting on race, ethnicity, or other socially relevant groupings	N/A
Population characteristics	N/A
Recruitment	N/A
Ethics oversight	N/A

Note that full information on the approval of the study protocol must also be provided in the manuscript.

Field-specific reporting

Please select the one below that is the best fit for your research. If you are not sure, read the appropriate sections before making your selection.

☐ Life sciences ☐ Behavioural & social sciences ☒ Ecological, evolutionary & environmental sciences

For a reference copy of the document with all sections, see nature.com/documents/nr-reporting-summary-flat.pdf

Ecological, evolutionary & environmental sciences study design

All studies must disclose on these points even when the disclosure is negative.

Study description	An investigation of the factors determining the outcomes of aggressive interactions at bird feeders throughout the United States and Canada, using behavioural data from a participatory science platform (FeederWatch) in combination with spatial overlap data from eBird and other ecological variables compiled from the literature, using phylogenetically informed statistical procedures.
Research sample	We analyzed 12,765 co-occurring species pairs in the full FeederWatch dataset (https://feederwatch.org/explore/raw-dataset-requests/), which were all species present at birdfeeders in the FeederWatch dataset during the sample period (2015-2020), and then in a subset without predatory species in which successful displacements occurred (1,664 pairs). This subset comprised 99 species from 63 genera as aggressive species, and 176 species from 97 genera as the targets of aggression. This data represents a diverse set of species from across multiple avian families.
Sampling strategy	Sample sizes were not pre-determined, but rather consisted of all observations available from the onset of the data collection period (Fall 2015) to the start of the present study (Spring 2020), comprising 88,988 interactions.
Data collection	Behavioural observations of birds at feeders were submitted by thousands of participatory scientists from across the United States and Canada through the FeederWatch program. Specifically, individuals were asked to record the number and species identity of birds at their feeders, as well as informations about observed displacements, including the identity of the winner and loser of each successful displacement.
Timing and spatial scale	Behavioral data was submitted from 2015-20202. The behavioral data is from feeders at sites across the United States and Canada.
Data exclusions	The analyses were run on the full dataset and subsets of data. The subsets of data excluded predator species and for certain analyses excluded species with no range overlap.
Reproducibility	As this was not an experiment or field test there were no attempts to reproduce the experiment. However, the same qualitative results are retrieved with different subsets of data.

Randomization	As this was not an experimental study there was no randomization. When analyzing competitive interactions across species one must control for evolutionary distance and body mass. We include those covariates in the statistical models when analyzing competitive outcomes.
Blinding	Although there was no formal blinding, the observers submitting data and interactions had no knowledge of the questions that would be asked with the data. Therefore the individuals that submitted data could not have subconsciously tilted the results or outcomes.

Did the study involve field work? ☐ Yes ☒ No

Reporting for specific materials, systems and methods

We require information from authors about some types of materials, experimental systems and methods used in many studies. Here, indicate whether each material, system or method listed is relevant to your study. If you are not sure if a list item applies to your research, read the appropriate section before selecting a response.

Materials & experimental systems

n/a	Involved in the study
☒	☐ Antibodies
☒	☐ Eukaryotic cell lines
☒	☐ Palaeontology and archaeology
☒	☐ Animals and other organisms
☒	☐ Clinical data
☒	☐ Dual use research of concern
☒	☐ Plants

Methods

n/a	Involved in the study
☒	☐ ChIP-seq
☒	☐ Flow cytometry
☒	☐ MRI-based neuroimaging

Plants

Seed stocks	N/A
Novel plant genotypes	N/A
Authentication	N/A
