# Supplementary material for: Unfamiliarity generates costly aggression in interspecific avian dominance hierarchies
Source: Nat Commun. 2024 Jan 6;15:335. doi: 10.1038/s41467-023-44613-0 (PMC10771497; doi:10.1038/s41467-023-44613-0)
Supplement: Supplementary file 2 — Peer Review File [file 41467_2023_44613_MOESM2_ESM.pdf]

Peer Review File

Unfamiliarity generates costly aggression in interspecific avian dominance hierarchies

Open Access This file is licensed under a Creative Commons Attribution 4.0 International License, which permits use, sharing, adaptation, distribution and reproduction in any medium or format, as long as you give appropriate credit to the original author(s) and the source, provide a link to the Creative Commons license, and indicate if changes were made. In the cases where the authors are anonymous, such as is the case for the reports of anonymous peer reviewers, author attribution should be to 'Anonymous Referee' followed by a clear attribution to the source work. The images or other third party material in this file are included in the article's Creative Commons license, unless indicated otherwise in a credit line to the material. If material is not included in the article's Creative Commons license and your intended use is not permitted by statutory regulation or exceeds the permitted use, you will need to obtain permission directly from the copyright holder. To view a copy of this license, visit http://creativecommons.org/licenses/by/4.0/.

Reviewers' Comments:

Reviewer #1:
Remarks to the Author:
This MS is a really interesting extension of the earlier work from this team (Behav Ecol 2017), which set the groundwork for the current MS. I really like how the current MS carefully considers sympatry/synopy of the species pairs, and their potential vs actual pairwise interactions. I find the results quite exciting and think that the striking results and the breadth of species included in the analysis will make the paper exciting for a broad readership. That said, there are always areas for improvement, which I think will make the paper stronger. In my view, these suggestions are feasible to accomplish and are unlikely to impact the main analyses/results.

I have two areas where improvement would make the paper much easier to understand and would clarify some areas of confusion and terminology for me:

1. There are several sections that need clarification in the methods.

- For example, L268 explains the calculation of DEE, but I can't tell if this is calculated per region, feeder, or the whole dataset. Or is this based on L261, where species must have been observed aggressing at least once to be included in the dataset that was then simulated? Please clarify this section.

- L278: Why was this measure of directionality chosen? What are the benefits of this method versus other commonly-used methods? Also, please consider more neutral terms here, like "wins" and "losses" rather than dominant and subordinate. Usually in dominance research, it's more typical to summarize the raw win/loss interaction data into a categorical description of the dominance relationship. Here, n dominant and n subordinate are used for the raw numbers of wins and number of losses, which I find somewhat confusing. If you want to stick with these terms, I think describing first how you determined the categorical relationships (which is dominant, which is subordinate) would really clarify this section.

- L298: Once these characteristics were shared or not between species pairs, what is done with that comparison? Is there some kind of weighted similarity measure? Or is each variable categorical ("same" vs "different" diet, etc)? I would guess these measures would be continuous, but more information is needed here to follow the methods. The way they seem to be used in Figure 1 suggests they might be binary? I really can't tell – please add clarification to this section.

2. There is a mismatch for me between wording the approach as a "dominance hierarchy" versus dyadic dominance relationships. Here, the authors have not actually made a full dominance hierarchy (or if they did, it's unclear what methods they used). It would be much more appropriate to think of this as dyadic dominance or species dominance relationships, especially as the species pairs possibilities are going to shift regionally and by habitat. This is especially important to consider carefully in the interpretation of the results. For example L178 states "We also found striking support for the hypothesis that familiarity plays a key role in the formation and maintenance of interspecific dominance hierarchies. Species that are unfamiliar with local hierarchies engage in more frequent and less structured aggressive interactions at feeders, increasing the time and energy spent in such agonistic encounters." For me, the results that if two species aren't frequently found together that they have a less-defined dominance relationship is sufficiently interesting. It's not unfamiliarity with the whole dominance hierarchy (all potential species pairs and the rank-order of their dominance) – it's really potential unfamiliarity with that particular dyadic relationship(s) that are the focus of all the analyses. Please carefully consider the terminology used to avoid confusion.

General comments:

- Methods, general comments: I find it very helpful in the methods to stress both what was done, but also why each thing was done/included. I like to have an introductory sentence for many parts of the methods to make it easier for the reader to see why each part of the methods was done and how it fits in with the goals of the paper. For example, L336 (Phylogeny section), could you add something that reminds readers what you are using the phylogeny data for? Something like: "To control our analyses of species aggression by phylogenetic relatedness, we built a phylogeny for the species in our dataset".

- It would be great to see more incorporation of some of the recent mixed-species literature, especially about how species may tell each other apart (eg Ferdinand et al.) and how dominance might operate in mixed species groups (eg Coppinger et al.)

- The MS shifts unpredictably between past and present tense – I would recommend using past tense for all analyses as well as interpretations of the results and making all tenses consistent across the MS. I usually prefer present for on-going interactions, simulation results, or patterns that are extremely well-supported by multiple studies and likely generalized. I think here it would be clearer and safer to use past tense for most everything.

Line comments:

L146: The section header really emphasizes the "unfamiliarity" result, but this section also includes information on when other characteristics differ (relatedness, body size). Why are all of these results grouped in this section? Please consider changing the section title from "unfamiliarity" to something like "dissimilarity".

L177-178: There is a typo in this sentence: "Our analyses provide further evidence that aggressive interspecific interactions are more likely [to] arise between ecologically similar species"

L186-194: This discussion would benefit from including some recent interspecific work on species recognition and attraction/repulsion, for example Ferdinand et al.

L178-182: This section is worded much more strongly than I think is warranted from the results. "We also found striking support for the hypothesis that familiarity plays a key role in the formation and maintenance of interspecific dominance hierarchies. Species that are unfamiliar with local hierarchies engage in more frequent and less structured aggressive interactions at feeders, increasing the time and energy spent in such agonistic encounters.

L220: Inclusion of recent ideas about interspecific aggression among known individuals might be helpful to include here (eg Coppinger et al.), especially if relationships form between specific individuals across species boundaries in ways that can reverse general expectations of species dominance relationships.

L238: What is the justification for removing single observations of species pairs, rather than "rare" observations (which could be more than just a single observation)? Please add information here about why 1 obs was chosen as the threshold for exclusion.

L248: This section might be a good way to start the results section, as a good introduction to the breadth of species in the database.

Figure 1: Please add either a key or text describing what the black and grey colors mean (I'm guessing grey points are not statistically significant?) If this is the case, it looks like there's space here to add that information, maybe across the top of each panel? Also, I can't figure out the x-axis category orders. Can these get ordered by "topic" to improve readability? It would be great for

example to have sympatry and syntopy next to each other, and maybe DEE all the way to the right-side (especially as DEE is a main highlight, right? Where it is now it's getting a bit buried in the details).

Figure 2: I really like this figure! I have a couple suggestions for making it a bit clearer. The phylogeny is current very light grey and is hard to see in the background, possibly making it darker might improve visibility? For the two example edges, I find the dotted lines hard to see. What about a thicker line? And/or one in the same color family, but slightly brighter than the rest of the edges? I like the example pair images, but for clarity, can you add a leader line or something to more clearly indicate their nodes?

Figure 4?: I feel like one additional figure would be really helpful to tie together some of the main results of the paper. Could you do some kind of alluvial plot to show how all the factors you analyzed are linked?

Refs:

B. A. Coppinger, N. V. Carlson, T. M. Freeberg and K. E. Sieving. 2023. Mixed-species groups and the question of dominance in the social ecosystem. Philosophical Transactions of the Royal Society B: Biological Sciences Volume 378, Issue 1878 https://doi.org/10.1098/rstb.2022.0097

Ferdinand V, Pattenden E, Brightsmith DJ, Hobson EA. Inferring the decision rules that drive co-foraging affiliations in wild mixed-species parrot groups. Philosophical Transactions of the Royal Society B. 2023 Jun 5;378(1878):20220101. https://doi.org/10.1098/rstb.2022.0101

Reviewer #2:
Remarks to the Author:
The authors use an extensive dataset on aggressive interactions among bird species to test the factors that predict the occurrence of aggression among species, the incidence of aggression among species controlling for abundance, and the directionality or asymmetry of the interactions among species, with a focus on degree of familiarity among species as a predictor. The data are compiled from a large community science initiative where birds are interacting in a common setting (bird feeders during the nonbreeding season), replicated across a large part of North America. The authors find that interspecific aggression is more frequent among ecologically similar species, and involving species that are more aggressive with members of their own species. Species that cooccur in the same habitat have more consistent dominance hierarchies and increased likelihood of aggression, but reduced aggression controlling for abundance. The authors also find that interactions are less common and more directional (asymmetric) among species that differ more in size, and that interactions are more symmetric and less common than expected given abundance among more distantly related species. Overall, the study supports the ideas that aggression among species is adaptive, and that dominance hierarchies and familiarity lead to decreases in overt aggression.

The writing was mostly clear, as were the figures. I was most impressed with the careful and detailed measures incorporated into the analysis (e.g., rates of aggression that controlled for relative abundance, detailed quantitative estimates of sympatry and syntopy using eBird checklists) that give me confidence in the results. The overall results are significant - an increasing number of studies report that interference among species is more widespread and important than previously thought, and this study provides some of the strongest evidence to address central ideas about when and why aggressive interactions occur. My main comments on the manuscript (detailed below) center on clarity - I had a bit of trouble figuring out the details of the interaction data and how they relate to the main response measures.

Main comments:

lines 116-123 & Methods: As best I can tell, you have data on which species initiated each aggressive encounter, and which species was the target of the aggression, but I can't tell if you have data on the outcome (who won, or if the outcome was a draw). Did aggressive interactions include aggressive signalling (without physical aggression) as well? I'm guessing that more subtle interactions were not included (e.g., a subordinate retreats before the dominant arrives at the feeder)?

lines 123-128: "Similarly, with increasing species familiarity, interactions between species would be resolved towards the dominant member of the pair more than expected."
I would expect that individuals would be more likely to make mistakes when they are unfamiliar with the other species, where 'mistakes' are revealed by individuals who attack another species but then lose the fight. Are your data detailed/refined enough to test this idea? In some ways, this would be a stronger test of some of the ideas related to the costs of aggression and the importance of familiarity and established dominance hierarchies.

lines 284-286: "Ndominant is the number of displacements initiated by the species in the pair which initiates the highest number of displacements, and Nsubordinate is the number of displacements initiated by the species in the pair which initiates the fewest number of displacements." In my experience, dominant and subordinate are more often defined by the outcomes of the interactions - who wins or loses - rather than which species initiates the interactions. I presume that the initiator of the interaction usually won? Do you have data to support this? Statements like lines 126-128 (quoted above; "... interactions between species would be resolved towards the dominant member ...") suggest that you are using data on the outcomes of interactions rather than who initiated them, but I don't see outcome data mentioned in the Methods.

Minor comments:

Generally, I had trouble keeping each measure straight in my head as I read through the manuscript. It would help if you used simple and consistent names for each measure every time that you discuss them and retained the same order for presenting the different metrics. For example, Figure 1 has a panel for "Displacement observed" - it would be nice to have that same term used in your first description (lines 116-117). The order of measures in Figure 1 differs from the order in other parts of the manuscript (e.g., lines 116-123). Also, supplemental figures refer to 'SES' which (I think) is the same as 'DEE' in the main manuscript. Please use consistent notation / clarify. (Same for the dataset, which uses SES)

lines 94-96: "to do so individuals recognize salient cues." Increasing evidence that they are using signals, and that they accentuate or highlight these in aggressive encounters (e.g., https://peerj.com/articles/13431/).

lines 177-178: " are more likely arise ..." typo

lines 242-245: I liked the removal of predators and singleton pairs for the main analysis - makes sense.

lines 270-273: I really liked the DEE measure that controls for abundance using simulations to create a null expectation.

lines 294-296: Given that interactions are occurring (primarily) during the non-breeding seasons, why include the type of breeding territory defended by each species? Why not include information on whether the species defends wintering territories instead?

Figure 3: (B) and (C) in the legend appear to be reversed in the panels.

I couldn't run the R code without the phylogenetic tree (which I did not see among the supplementary files), but the code looked fine, and the dataset looked complete.

Reviewer #3:
Remarks to the Author:
I read this paper with great interest given my interest in the effects of interspecific aggression on bird communities and also from my own experience with birds interacting aggressively at point sources of food. In some respects, the resulting manuscript was even better than I had anticipated with strong hypotheses and alternative hypotheses, innovative analytical tools, an even larger data set than I was aware of, and conclusions that seem consistent with my own experience. Indeed I have few comments of an editorial nature.

My main issue with the manuscript is that there is no real discussion of the context in which all of these data were gathered--bird feeders. As the authors know well, bird feeders are something unusual in nature--a permanent point source of renewing food that promotes aggressive defense, both intra- and interspecific. Such resources are probably rare in nature, although interspecific hierarchies have been observed in fruiting trees and around ant swarms. The unusual concentration of food resources at bird feeders does not invalidate your results; I am sure that the hierarchies observed at feeders are similar to those in other ecological contexts. But, I do think that increasing urbanization with its increasing number of feeders and point sources of food such as at dumps may tend to favor interpecifically dominant species. I would like to see an additional paragraph in the discussion dealing with the possibility that the results from these studies of bird feeders have great relevance for the future of bird communities in an urbanizing world. I would also like to see some mention of how invasive species and new species combinations that are created by urbanization and the spread of invasive species might destabilize these hierarchies.

One final point I would like to see addressed is how social behavior, both intra-specific and inter-specific, may affect these results. Species that tend to occur in flocks can overcome interspecific aggression through sheer numbers. Dominant species might avoid interacting with smaller species that live in groups for the same reason that they avoid costly fights with larger species; it is just too costly to chase away a whole flock of individuals even if they are subordinate. The feeders may also promote aggressive interactions among species that may co-occur peacefully in mixed-species flocks where they are usually exploiting less concentrated food resources and their foraging niches may be different enough to minimize competition. In any case, flocking will certainly affect which species tend to occur together and familiarity among some species pairs.

Response to Reviewers

>> We thank the reviewers for their constructive feedback that has improved the quality of the manuscript. We address and incorporate virtually all suggestions from the reviewers, and in any rare case where we do not follow the specific suggestion we provide a rationale for our decision. All responses begin with double arrows ">>" to signify our response and are color coded purple to differentiate responses from reviewer suggestions.

REVIEWER COMMENTS

Reviewer #1 (Remarks to the Author):

This MS is a really interesting extension of the earlier work from this team (Behav Ecol 2017), which set the groundwork for the current MS. I really like how the current MS carefully considers sympatry/synopy of the species pairs, and their potential vs actual pairwise interactions. I find the results quite exciting and think that the striking results and the breadth of species included in the analysis will make the paper exciting for a broad readership. That said, there are always areas for improvement, which I think will make the paper stronger. In my view, these suggestions are feasible to accomplish and are unlikely to impact the main analyses/results.

>> We thank the reviewer for their supportive feedback.

I have two areas where improvement would make the paper much easier to understand and would clarify some areas of confusion and terminology for me:

1. There are several sections that need clarification in the methods.

- For example, L268 explains the calculation of DEE, but I can't tell if this is calculated per region, feeder, or the whole dataset. Or is this based on L261, where species must have been observed aggressing at least once to be included in the dataset that was then simulated? Please clarify this section.

>> The simulation data are generated/sampled at each feeder and then aggregated across all feeders in each iteration of the simulation. The DEE calculation is not restricted to species who were observed to interact—all species present in the FeederWatch checklists are incorporated into the simulation analysis (though probabilities of interaction at each feeder only incorporate species present at that feeder). We have now added more detail that explains this (rather than simply referring to the methods in a previous publication) at lines 350-355, which we hope improves the clarity.

- L278: Why was this measure of directionality chosen? What are the benefits of this method versus other commonly-used methods? Also, please consider more neutral terms here, like "wins" and "losses" rather than dominant and subordinate. Usually in dominance research, it's more typical to summarize the raw win/loss interaction data into a categorical description of the dominance relationship. Here, n dominant and n subordinate are used for the raw numbers of wins and number of losses, which I find somewhat confusing. If you want to stick with these

terms, I think describing first how you determined the categorical relationships (which is dominant, which is subordinate) would really clarify this section.

>> We have edited the language in this section to clarify that we are analyzing individual wins and losses. We now use 'wins by dominant' and 'wins by subordinate' (as used in Martin & Ghalambor 2014) to clarify that the raw win/loss interaction data are used first to determine which of the two interactors are dominant (i.e., which one 'wins' the most frequently).

Although the reviewer references other methods to quantify inequality in wins and losses between species, we argue that the method used in the current manuscript sufficiently captures biological dominance for the questions at hand. As an example, we have identified a set of species pairs where the Tufted Titmouse is included in each pair to support our point:

sp1	sp2	sp1.win	sp2.win	directionality
Tufted titmouse	Brown-headed nuthatch	6	6	0.000000
Tufted titmouse	American goldfinch	318	85	1.144921
Tufted titmouse	Northern mockingbird	0	156	2.248610

These represent low, medium, and high values of directionality and we argue that these are intuitive and realistic representations of these species pair interactions. We have therefore made some revisions to wording in the manuscript to be more consistent and clear with describing the Directionality variable.

- L298: Once these characteristics were shared or not between species pairs, what is done with that comparison? Is there some kind of weighted similarity measure? Or is each variable categorical ("same" vs "different" diet, etc)? I would guess these measures would be continuous, but more information is needed here to follow the methods. The way they seem to be used in Figure 1 suggests they might be binary? I really can't tell – please add clarification to this section.

>> These measures are categorical, and thus trying to assess each combination is would generate an inordinate amount of statistical comparisons. Consequently, we converted them into binomial variables. We have now explained this in the main text (line 390).

2. There is a mismatch for me between wording the approach as a "dominance hierarchy" versus dyadic dominance relationships. Here, the authors have not actually made a full dominance hierarchy (or if they did, it's unclear what methods they used). It would be much more appropriate to think of this as dyadic dominance or species dominance relationships, especially as the species pairs possibilities are going to shift regionally and by habitat. This is especially important to consider carefully in the interpretation of the results. For example L178 states "We also found striking support for the hypothesis that familiarity plays a key role in the formation and maintenance of interspecific dominance hierarchies. Species that are unfamiliar with local hierarchies engage in more frequent and less structured aggressive interactions at feeders, increasing the time and energy spent in such agonistic encounters." For me, the results that if two species aren't frequently found together that they have a less-defined dominance relationship is

sufficiently interesting. It's not unfamiliarity with the whole dominance hierarchy (all potential species pairs and the rank-order of their dominance) – it's really potential unfamiliarity with that particular dyadic relationship(s) that are the focus of all the analyses. Please carefully consider the terminology used to avoid confusion.

>> This is a fair point to raise, but we hope that we can explain our rationale for framing our analyses within the context of understanding the factors that shape interspecific dominance hierarchies. Firstly, previous analyses of the FeederWatch dataset clearly demonstrate (using standard analyses designed to reconstruct dominance) that the species at feeders are organised into a stable, interspecific dominance hierarchy. Our motivation, therefore, was to understand what factors influence the presence, strength, and directionality in these interspecific dominance hierarchies, and how they compare to factors known to influence intraspecific dominance hierarchies. While it is true that the analyses we conduct are pairwise, they incorporate all possible pairs and are not limited to sister taxa (a common approach for phylogenetic analyses of pairwise interactions), and therefore represent the strongest possible way (to our knowledge) to test hypotheses about individual links within dominance hierarchies. We have made several edits to the text in the hopes of making these points clearer (lines 110-115).

General comments:

- Methods, general comments: I find it very helpful in the methods to stress both what was done, but also why each thing was done/included. I like to have an introductory sentence for many parts of the methods to make it easier for the reader to see why each part of the methods was done and how it fits in with the goals of the paper. For example, L336 (Phylogeny section), could you add something that reminds readers what you are using the phylogeny data for? Something like: "To control our analyses of species aggression by phylogenetic relatedness, we built a phylogeny for the species in our dataset".

>> We have added a sentence to this effect in the manuscript (lines 438-439).

- It would be great to see more incorporation of some of the recent mixed-species literature, especially about how species may tell each other apart (eg Ferdinand et al.) and how dominance might operate in mixed species groups (eg Coppinger et al.)

>> We thank the reviewer for pointing us toward these excellent papers (and indeed, this special issue of Phil Trans). We have incorporated them in our revised manuscript (e.g., lines 78, 234, 277, 293,)

- The MS shifts unpredictably between past and present tense – I would recommend using past tense for all analyses as well as interpretations of the results and making all tenses consistent across the MS. I usually prefer present for on-going interactions, simulation results, or patterns that are extremely well-supported by multiple studies and likely generalized. I think here it would be clearer and safer to use past tense for most everything.

>> Thanks for pointing this out—we have fixed a few inconsistencies with tense in the Methods section. However, following guidance from the Editor regarding journal style, we have used

present tense to discuss the current work in the Abstract and Introduction, only using past tense to describe previously published findings.

Line comments:

L146: The section header really emphasizes the “unfamiliarity” result, but this section also includes information on when other characteristics differ (relatedness, body size). Why are all of these results grouped in this section? Please consider changing the section title from “unfamiliarity” to something like “dissimilarity”.

>> The author brings up a point worthy of consideration regarding the results section title. We have made a slight modification to the results section title, but retain the focus on unfamiliarity. Specifically, we add the other major predictors of the dependent variables to the results section titles. In doing so we highlight the other results but also maintain focus on the main connecting aspect throughout the results. We retain the term unfamiliarity in the section title because of the centrality of the syntopy results for the three focal dependent variables related to aggression. Losing the through line in the results could reduce reader understanding. As a result we are hesitant to alter the results section title in full.

L177-178: There is a typo in this sentence: “Our analyses provide further evidence that aggressive interspecific interactions are more likely [to] arise between ecologically similar species”

>>Thanks for catching this! Fixed.

L186-194: This discussion would benefit from including some recent interspecific work on species recognition and attraction/repulsion, for example Ferdinand et al.

>>Done. (Lines 233-234)

L178-182: This section is worded much more strongly than I think is warranted from the results. “We also found striking support for the hypothesis that familiarity plays a key role in the formation and maintenance of interspecific dominance hierarchies. Species that are unfamiliar with local hierarchies engage in more frequent and less structured aggressive interactions at feeders, increasing the time and energy spent in such agonistic encounters.

>>We have softened the language here (Lines 216-220).

L220: Inclusion of recent ideas about interspecific aggression among known individuals might be helpful to include here (eg Coppinger et al.), especially if relationships form between specific individuals across species boundaries in ways that can reverse general expectations of species dominance relationships.

>>We have included these recent ideas and publications in the text (Lines 293).

L238: What is the justification for removing single observations of species pairs, rather than

“rare” observations (which could be more than just a single observation)? Please add information here about why 1 obs was chosen as the threshold for exclusion.

>>We thank the reviewer for this comment and explain our rationale here and have added language in the text of the manuscript (lines 314-315). Specifically, we examined several extreme DEE (SES) scores and noticed they were due to species pairs that interacted only once at one feeder. We wanted to confirm that these extreme values with high statistical leverage did not influence the results; consequently, we removed them from the analysis. Importantly, the analyses of this subset of data are qualitatively identical with the results from the full dataset.

In biological terms these singleton observations between species pairs could represent situations where exceedingly rare ecological or behavioral factors drive the species to interact. We wanted to remain conservative and remove these cases from the dataset and only incorporate data from species pairs that have more consistent interactions.

L248: This section might be a good way to start the results section, as a good introduction to the breadth of species in the database.

>> We agree that this would be a helpful change and have implemented it (lines 134-144).

Figure 1: Please add either a key or text describing what the black and grey colors mean (I’m guessing grey points are not statistically significant?) If this is the case, it looks like there’s space here to add that information, maybe across the top of each panel? Also, I can’t figure out the x-axis category orders. Can these get ordered by “topic” to improve readability? It would be great for example to have sympatry and syntopy next to each other, and maybe DEE all the way to the right-side (especially as DEE is a main highlight, right? Where it is now it’s getting a bit buried in the details).

>> The reviewer is correct that the grey points represent effects that are not significant. We have added text to the figure caption to describe what grey and black represent in the figure.

We have now revised the figure to group spatial variables (sympatry and syntopy) and foraging variables. We have now placed other categorical variables in locations we think are as intuitive as possible.

We have moved the highest intraspecific DEE to the right in Figure 1. We have reorganized the predictor organization and also reorganized the vertical placement of panels following the suggestions of another reviewer.

Figure 2: I really like this figure! I have a couple suggestions for making it a bit clearer. The phylogeny is current very light grey and is hard to see in the background, possibly making it darker might improve visibility? For the two example edges, I find the dotted lines hard to see. What about a thicker line? And/or one in the same color family, but slightly brighter than the rest of the edges? I like the example pair images, but for clarity, can you add a leader line or something to more clearly indicate their nodes?

>>Thanks! We have thickened the dotted lines and changed their color, and we've added leader lines as suggested. We tinkered with the color of the phylogeny, but feel that darkening the color really decreases the readability of the plot.

Figure 4?: I feel like one additional figure would be really helpful to tie together some of the main results of the paper. Could you do some kind of alluvial plot to show how all the factors you analyzed are linked?

We thank the reviewer for their suggestion about a fourth figure. We attempted to workshop and generate rough drafts of a plot that resembles an alluvial plot. We produced the following as a figure that merges main results and the concepts of an alluvial plot:

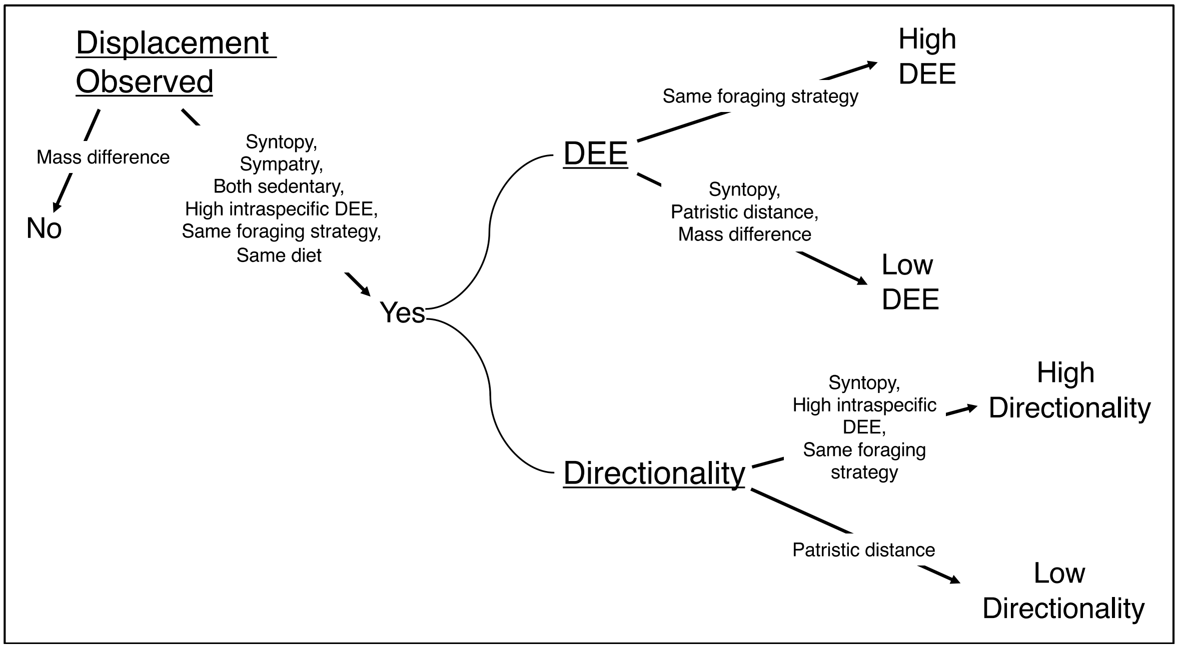

This figure attempts to connect main findings with the connection arrows typically found in alluvial plots. However, we remain unconvinced that our draft figure improves understanding. We are somewhat concerned that connecting arrows across individual variables might lead to confusion. For instance, in the figure above, a reader might assume that if displacement is observed then either DEE or Directionality is tested, rather than having variables tested across all data. We are therefore unsure about including a figure that would improve understanding above what readers can extract from figure 1. In figure 1 all the independent variables are aligned vertically, and thus readers can compare these effects quickly across all three variables.

Refs:

B. A. Coppinger, N. V. Carlson, T. M. Freeberg and K. E. Sieving. 2023. Mixed-species groups and the question of dominance in the social ecosystem. Philosophical Transactions of the Royal Society B: Biological Sciences Volume 378, Issue 1878 https://doi.org/10.1098/rstb.2022.0097

Ferdinand V, Pattenden E, Brightsmith DJ, Hobson EA. Inferring the decision rules that drive co-foraging affiliations in wild mixed-species parrot groups. Philosophical Transactions of the Royal Society B. 2023 Jun 5;378(1878):20220101. https://doi.org/10.1098/rstb.2022.0101

Reviewer #2 (Remarks to the Author):

The authors use an extensive dataset on aggressive interactions among bird species to test the factors that predict the occurrence of aggression among species, the incidence of aggression among species controlling for abundance, and the directionality or asymmetry of the interactions among species, with a focus on degree of familiarity among species as a predictor. The data are compiled from a large community science initiative where birds are interacting in a common setting (bird feeders during the nonbreeding season), replicated across a large part of North America. The authors find that interspecific aggression is more frequent among ecologically similar species, and involving species that are more aggressive with members of their own species. Species that cooccur in the same habitat have more consistent dominance hierarchies and increased likelihood of aggression, but reduced aggression controlling for abundance. The authors also find that interactions are less common and more directional (asymmetric) among species that differ more in size, and that interactions are more symmetric and less common than expected given abundance among more distantly related species. Overall, the study supports the ideas that aggression among species is adaptive, and that dominance hierarchies and familiarity lead to decreases in overt aggression.

The writing was mostly clear, as were the figures. I was most impressed with the careful and detailed measures incorporated into the analysis (e.g., rates of aggression that controlled for relative abundance, detailed quantitative estimates of sympatry and syntopy using eBird checklists) that give me confidence in the results. The overall results are significant - an increasing number of studies report that interference among species is more widespread and important than previously thought, and this study provides some of the strongest evidence to address central ideas about when and why aggressive interactions occur. My main comments on the manuscript (detailed below) center on clarity - I had a bit of trouble figuring out the details of the interaction data and how they relate to the main response measures.

>> We thank the reviewer for their supportive feedback.

Main comments:

lines 116-123 & Methods: As best I can tell, you have data on which species initiated each aggressive encounter, and which species was the target of the aggression, but I can't tell if you have data on the outcome (who won, or if the outcome was a draw). Did aggressive interactions include aggressive signalling (without physical aggression) as well? I'm guessing that more subtle interactions were not included (e.g., a subordinate retreats before the dominant arrives at the feeder)?

>> The overall FeederWatch dataset includes both successful and unsuccessful displacement attempts, though unsuccessful displacement attempts are a minority of submitted observations (~ 10%). Each of the attempts indeed has a source and target of aggression. However, we filter out any unsuccessful displacement attempts before analyzing the overall dataset and subsets of data (for several reasons described below). The dataset does not include information about signaling, nor details about species leaving. As the dataset is submitted by amateur ornithologists, the instructions for submitting interactions have been relatively simplified.

lines 123-128: "Similarly, with increasing species familiarity, interactions between species would be resolved towards the dominant member of the pair more than expected."
I would expect that individuals would be more likely to make mistakes when they are unfamiliar with the other species, where 'mistakes' are revealed by individuals who attack another species but then lose the fight. Are your data detailed/refined enough to test this idea? In some ways, this would be a stronger test of some of the ideas related to the costs of aggression and the importance of familiarity and established dominance hierarchies.

>> The reviewer highlights an interesting point regarding aggressive mistakes. With respect to the FeederWatch dataset, participants can and do submit what are known as 'Unsuccessful Displacements'. These unsuccessful displacements are analogous to the potential mistakes the reviewer refers to. However, these unsuccessful displacements are relatively rare in the datasets (~10%) of the observed interactions. This rarity is likely due to these types of interactions being biologically rare, and also an interaction that is more ambiguous, though their rarity may also result from the fact that communications around behavioural data submitted to FeederWatch have focused on successful displacements. Partially due to this rarity, the quality checks on these unsuccessful displacement interactions are not as stringent as the quality checks on the successful displacements. Consequently, previous publications and this manuscript have focused on employing the successful displacements in the analyses, for which we can be more confident that the spatial variation we observe is a reliable sample for analyzing the questions we pose in the manuscript.

However, these hesitations about 'unsuccessful' displacement data quality notwithstanding, we did utilize unsuccessful displacements to investigate the potential mistakes between species. To investigate unsuccessful displacements we performed several calculations for species pairs. First, we calculated the number of total displacements between species and removed species pairs that had fewer than 5 total displacements. We removed these pairs because in calculating the proportion of aggression that is unsuccessful, the proportion can shift considerably with only 3-4 interactions among species. The resulting data subset was zero inflated, so we analyzed the data in two ways. First, we determined whether all displacements between species pairs were successful (analyzed as a binomial variable), and then among species pairs where unsuccessful displacement was observed, which variables might explain the extent of unsuccessful displacement (analysed as a continuous variable).

Like successful displacements, we find that increasing syntopy between species leads to an increased chance of observing any unsuccessful displacement (posterior mean = -0.263, p = 0.04). We suspect that since this is a relatively rare type of interaction, species that come into contact more often are more likely to have been observed in an unsuccessful displacement. We

also find that with increasing sympatry between species we find a lower proportion of unsuccessful attacks (posterior mean = 0.027, p = 0.004), though we find no effect of syntopy on proportion of unsuccessful attacks. Thus, there is some suggestive (though not conclusive) evidence that species that have reduced range overlap might engage in more unsuccessful displacements.

While this evidence is intriguing, we have chosen not to incorporate the results into the current revision for a couple important reasons. The foremost reason is that the unsuccessful data has not been as intensively curated as the displacement data. Although the unsuccessful displacements are intriguing, we do not have as much confidence in these observations as the displacements. Second, the unsuccessful displacement data has yielded some interesting trends, but likely requires a concerted focus that is beyond the scope of the current manuscript. For instance, individuals might employ the unsuccessful data to see if unsuccessful displacements provide an inverse set of aggressive outcomes compared to successful displacements. In sum, we think the reviewer has highlighted an interesting question but we argue that a full investigation of this data is warranted in a future study in which FeederWatch participants are specifically briefed on the collection of unsuccessful displacement data.

lines 284-286: "Ndominant is the number of displacements initiated by the species in the pair which initiates the highest number of displacements, and Nsubordinate is the number of displacements initiated by the species in the pair which initiates the fewest number of displacements." In my experience, dominant and subordinate are more often defined by the outcomes of the interactions - who wins or loses - rather than which species initiates the interactions. I presume that the initiator of the interaction usually won? Do you have data to support this? Statements like lines 126-128 (quoted above; "... interactions between species would be resolved towards the dominant member ...") suggest that you are using data on the outcomes of interactions rather than who initiated them, but I don't see outcome data mentioned in the Methods.

>> Following this comment and a similar point made by the first reviewer, we have modified the text throughout to clarify how interactions were comprised of “winners” and “losers”, and how these outcomes were aggregated to determine the identify of dominant and subordinate species within species pairs.

The reviewer is correct that the individual that initiates the interaction wins in a large majority of cases (~83%) in the overall FeederWatch dataset. For our analyses we filter out unsuccessful displacements before running the analyses so that the $N_{\text{wins by dominant}}$ and $N_{\text{wins by subordinate}}$ variable values can be calculated following Martin and Ghalambor (2014). We have added text to the methods (lines 303-305) specifying that the interaction dataset we employed focused on successful displacements, and thus the source of the aggression succeeded and the target was forced from the resource.

Minor comments:

Generally, I had trouble keeping each measure straight in my head as I read through the manuscript. It would help if you used simple and consistent names for each measure every time

that you discuss them and retained the same order for presenting the different metrics. For example, Figure 1 has a panel for "Displacement observed" - it would be nice to have that same term used in your first description (lines 116-117). The order of measures in Figure 1 differs from the order in other parts of the manuscript (e.g., lines 116-123). Also, supplemental figures refer to 'SES' which (I think) is the same as 'DEE' in the main manuscript. Please use consistent notation / clarify. (Same for the dataset, which uses SES)

>> Following the suggestion of the reviewer we have added the specific name of the variable (Displacement observed) to the end of the introduction (Lines 117-118).

We have now modified Figure 1 following the suggestion of the reviewer, with the vertical order of the variables matching their order at the end of the introduction.

The reviewer is correct that DEE is identical to SES, we have updated the supplemental figures to update all references to DEE. We have also updated the submitted data to refer to DEE.

lines 94-96: "to do so individuals recognize salient cues." Increasing evidence that they are using signals, and that they accentuate or highlight these in aggressive encounters (e.g., https://peerj.com/articles/13431/).

>> Thanks for drawing our attention to this—we have now cited it (Lines 94-95).

lines 177-178: " are more likely arise ..." typo

>> We have corrected the typo

lines 242-245: I liked the removal of predators and singleton pairs for the main analysis - makes sense.

>> We thank the reviewer for this comment.

lines 270-273: I really liked the DEE measure that controls for abundance using simulations to create a null expectation.

>> We thank the reviewer for this comment.

lines 294-296: Given that interactions are occurring (primarily) during the non-breeding seasons, why include the type of breeding territory defended by each species? Why not include information on whether the species defends wintering territories instead?

>>Thank you for pointing this out. In fact, although we initially considered including breeding territory behavior at an early point, we had removed these data for this very reason, but failed to remove this text in the Methods. We have now removed this text. We haven't included information on wintering territories because, unfortunately, data on winter territories is very sparse (e.g., few species accounts in Birds of the World contain any information on winter territorial behavior).

Figure 3: (B) and (C) in the legend appear to be reversed in the panels.

>>Thank you for catching this! We have fixed the caption.

I couldn't run the R code without the phylogenetic tree (which I did not see among the supplementary files), but the code looked fine, and the dataset looked complete.

>>Good point—we have now included the phylogenetic tree in the supplementary materials.

Reviewer #3 (Remarks to the Author):

I read this paper with great interest given my interest in the effects of interspecific aggression on bird communities and also from my own experience with birds interacting aggressively at point sources of food. In some respects, the resulting manuscript was even better than I had anticipated with strong hypotheses and alternative hypotheses, innovative analytical tools, an even larger data set than I was aware of, and conclusions that seem consistent with my own experience. Indeed I have few comments of an editorial nature.

>> We thank the reviewer for their positive feedback.

My main issue with the manuscript is that there is no real discussion of the context in which all of these data were gathered--bird feeders. As the authors know well, bird feeders are something unusual in nature--a permanent point source of renewing food that promotes aggressive defense, both intra- and interspecific. Such resources are probably rare in nature, although interspecific hierarchies have been observed in fruiting trees and around ant swarms. The unusual concentration of food resources at bird feeders does not invalidate your results; I am sure that the hierarchies observed at feeders are similar to those in other ecological contexts. But, I do think that increasing urbanization with its increasing number of feeders and point sources of food such as at dumps may tend to favor interpecifically dominant species. I would like to see an additional paragraph in the discussion dealing with the possibility that the results from these studies of bird feeders have great relevance for the future of bird communities in an urbanizing world. I would also like to see some mention of how invasive species and new species combinations that are created by urbanization and the spread of invasive species might destabilize these hierarchies.

>> We thank the reviewer for the interesting ideas regarding urbanization, dominance hierarchies, and facilitation of non-native species. We have now added a paragraph to the discussion that incorporates these points and suggest that increasing urbanization might facilitate the invasion of non-natives, and thereby alter the dominance hierarchy (lines 260-273).

One final point I would like to see addressed is how social behavior, both intra-specific and inter-specific, may affect these results. Species that tend to occur in flocks can overcome

interspecific aggression through sheer numbers. Dominant species might avoid interacting with smaller species that live in groups for the same reason that they avoid costly fights with larger species; it is just too costly to chase away a whole flock of individuals even if they are subordinate. The feeders may also promote aggressive interactions among species that may co-occur peacefully in mixed-species flocks where they are usually exploiting less concentrated food resources and their foraging niches may be different enough to minimize competition. In any case, flocking will certainly affect which species tend to occur together and familiarity among some species pairs.

>> We thank the reviewer for the suggestions regarding sociality and the influence on competition and aggression. We have now added a paragraph to the discussion that highlights some of the findings regarding how sociality affects individuals and groups in dominance interactions (lines 275-285).

Reviewers' Comments:

Reviewer #1:
Remarks to the Author:
The authors have addressed my previous comments and I am satisfied with their revised version. I think this paper will be exciting to people working in a range of fields, from dominance to species range expansions, to invasive species effects, to climate change, etc.

Reviewer #2:
Remarks to the Author:
Thanks to the authors for addressing my comments/concerns.

A couple minor points:

Figure 1 caption needs updating to match the current layout of the panels (A,B,C).

Figure 3C. The model-predicted line seems offset from the datapoints (shifted upwards) - worth double-checking (e.g., that they are on the same scale). (also Suppl Fig 2B)

A very exciting paper!!

Reviewer #3:
Remarks to the Author:
The authors have addressed my two main concerns, both of which involved the discussion. The two paragraphs added deal with these issues well and should increase the interest generated by this paper, which reflects the reality of human-dominated landscapes: permanent point sources of food where these aggressive interactions will become increasingly important.

REVIEWERS' COMMENTS

Reviewer #1 (Remarks to the Author):

The authors have addressed my previous comments and I am satisfied with their revised version. I think this paper will be exciting to people working in a range of fields, from dominance to species range expansions, to invasive species effects, to climate change, etc.

Response: We thank the reviewer for their helpful feedback and positive response to our manuscript.

Reviewer #2 (Remarks to the Author):

Thanks to the authors for addressing my comments/concerns.

A couple minor points:

Figure 1 caption needs updating to match the current layout of the panels (A,B,C).

Response: Thank you, fixed.

Figure 3C. The model-predicted line seems offset from the datapoints (shifted upwards) - worth double-checking (e.g., that they are on the same scale). (also Suppl Fig 2B)

Response: Thank you for pointing this out--we agree this is unexpected. We carefully examined this issue, and believe that it may come down to issues with the scripts used to predict model values using the model fits from the R package MCMCglmm (e.g., in the "Course Notes" [dated October 2017] describing this package, the author writes on p. 47, " A predict function has been implemented for MCMCglmm although it is currently incomplete and needs further testing."). For ease of visualisation, therefore, we have plotted the relationships predicted from simple GLM fits, which we explain in our figure captions.

A very exciting paper!!

Response: We thank the reviewer for their helpful feedback and positive response to our manuscript.

Reviewer #3 (Remarks to the Author):

The authors have addressed my two main concerns, both of which involved the discussion. The two paragraphs added deal with these issues well and should increase the interest generated by this paper, which reflects the reality of human-dominated landscapes: permanent point sources of food where these aggressive interactions will become increasingly important.

Response: We thank the reviewer for their helpful feedback and positive response to our manuscript.
